# Pharmacokinetics and metabolic effects of ketone monoester supplementation: The first simultaneous CKM and CGM study under normal diet and activities

**DOI:** 10.1016/j.metop.2025.100411

**Published:** 2025-10-29

**Authors:** Toshiya Miyatsu, Connor Tate, Jeremy McAdam, Chandler Massey, Timothy Broderick

**Affiliations:** Institute for Human & Machine Cognition, USA

## Abstract

Exogenous ketone supplementation has gained attention for its potential health and performance benefits, yet its real-world pharmacokinetics and metabolic effects remain underexplored. This study investigated the pharmacokinetics of ketone monoester (KME) supplementation during normal diet and activities, leveraging simultaneous interstitial fluid (ISF) continuous ketone and glucose monitoring (CKM and CGM). In this single-group observational study, twenty healthy adults underwent a 10-day KME supplementation protocol following a 4-day baseline period. Weight-based KME dosing rapidly elevated ISF β-hydroxybutyrate (BHB) levels, peaking within 1 h and sustaining ketosis for approximately 5 h. Exploratory correlational analyses revealed a two-stage influence pattern: larger skeletal/adipose mass slowed and blunted peak BHB levels (*r* = −.52 to −.63, *p* = .04–.08), whereas higher habitual activity, better baseline glucose regulation and greater protein intake prolonged BHB elevation and associated glucose-suppression window (*r* = .47–.58, *p* = .05–.09). Granger causality analysis confirmed that KME supplementation acutely suppressed ISF glucose, with an initial effect at 5 min and a sustained post-dose suppression phase between 25 and 55 min. Surprisingly, fasting glucose levels increased after 10 days of KME supplementation, suggesting compensatory metabolic adaptation. Additionally, sleep efficiency and quality declined during the intervention phase. These findings highlight the complex metabolic effects of KME use, emphasizing the need for personalized approaches to optimize its benefits. This study demonstrates the feasibility of CKM/CGM for capturing real-time metabolic dynamics and underscores the importance of further research into the physiological implications of exogenous ketone supplementation.

The classic ketogenic diet, a low-carbohydrate, high-fat regimen that induces hepatic production of ketone bodies, has long been used for epilepsy and is now being explored for metabolic regulation, weight management, and performance [[1], [2], [3]]. Despite its benefits, strict dietary adherence is difficult, creating demand for alternative strategies such as exogenous ketone supplementation [4]. The therapeutic potential of exogenous ketones has garnered significant interest across multiple clinical and performance domains, from neuroprotection [5,6] to metabolic regulation [4,7] to endurance performance enhancement [8,9]. Among available ketone supplements, ketone monoesters (KME) have emerged as particularly promising due to their ability to rapidly and effectively elevate blood β-hydroxybutyrate (BHB) concentrations. (R)-β-Hydroxybutyrate-(R)-1,3 Butanediol represents an important advance in this field as a generally recognized as safe (GRAS) food supplement that can rapidly achieve therapeutic levels of ketones in blood (>.5 mmol/L). Unlike endogenous ketones generated with a ketogenic diet, exogenous monoesters deliver pre-formed BHB directly into circulation, bypassing hepatic β-oxidation and enabling dose-response control [10].

Recent research has demonstrated the safety and tolerability of KME in controlled laboratory settings. These studies have typically involved fasted subjects or standardized meal protocols, providing valuable initial pharmacokinetic data [10]. However, the behavior of ketone supplements during normal diet and activities - the conditions under which they would most likely be used - remains poorly characterized. Understanding ecologically valid pharmacokinetics is essential for developing evidence-based supplementation protocols that optimize clinical outcomes, health and performance.

Continuous analyte monitoring has transformed metabolic research and diabetes care [11]. The recent development of interstitial fluid (ISF) continuous ketone monitoring (CKM) technology represents a significant methodological advance for studying ketone dynamics [12,13]. Widely used continuous glucose monitors (CGMs) are based upon an implanted electro-enzymatic filament with glucose oxidase on a platinum working electrode to convert local ISF glucose into an electrical current that is temperature-compensated and algorithmically translated into glucose values every 1–5 min [14]. Next-generation CKMs employ a similar architecture, substituting BHB-specific dehydrogenase to oxidize BHB and provide ISF ketone levels [12]. Both CGM and CKM now demonstrate clinically acceptable precision. Factory-calibrated CGM routinely achieve a mean absolute relative difference (MARD) of 8–10 % versus venous plasma glucose, comparable to modern point-of-care glucose meters [15]. Early validation of the Abbott Keto Lingo CKM showed a MARD of ∼14 % and a Pearson *r* > .90 relative to capillary BHB [12], indicating sufficient precision for research and therapeutic monitoring. Emerging continuous lactate monitors (CLMs) could extend the platform to additional metabolites [16].

Traditional ketone level assessment relies on intermittent sampling of venous blood through phlebotomy or capillary blood through finger prick, which limit the temporal resolution of pharmacokinetic data and pose practical challenges for extended monitoring periods. ISF BHB monitoring enables continuous assessment of ketone levels in one's daily life. Because ketone levels are closely related to glucose levels, combining CKM with CGM offers dual-analyte perspective on substrate competition and metabolic flexibility that cannot be achieved with either modality alone.

To our knowledge, this is the first study to deploy simultaneous CKM and CGM under free-living conditions, thereby enabling high-resolution mapping of exogenous ketone pharmacokinetics alongside real-time glycemic responses. The current study leverages simultaneous analyte monitoring to comprehensively characterize the pharmacokinetics of KME supplementation during normal diet and activities. We established ecologically valid pharmacokinetics for weight-stratified dosing to facilitate translation into occupational and clinical settings. This design addresses practical challenges in ketone supplementation protocols where interindividual variability in body composition, diet and activity can significantly impact ketone level and metabolic response. Secondary aims include investigation of relationships between KME supplementation and glycemic variability.

## 1 Methods

**Design**. We employed a single-group observational design consisting of a 4-day baseline phase followed by a 10-day intervention phase involving KME supplementation. During the baseline phase, participants wore all monitoring devices under normal diet and activity to establish their habitual metabolic, dietary, and sleep profiles. This baseline provided individualized reference values for subsequent comparisons during the supplementation phase. A 4-day duration was chosen because prior free-living continuous monitoring studies have shown that 3–5 days of CGM or actigraphy are sufficient to capture stable individual patterns in glucose regulation and sleep behavior [11,17]. During the intervention phase, participants continued wearing all devices while receiving twice-daily, weight-stratified KME doses.

**Participants**. Twenty healthy young adults (10 males and 10 females) aged 18–35 were recruited. Exclusion criteria include history of metabolic disorders, ketogenic diet, diabetes, pregnancy, or other conditions interfering with study outcomes. The study protocol received approval from the Institute for Human & Machine Cognition (IHMC) Institutional Review Board (IRB-2022-0040) and was conducted in compliance with rules, regulations, and ethical standards for research involving human subjects. The recruitment and data collection were conducted at IHMC and remotely from January 10 to 25, 2024. The sample size was based on a previous study of similar kind.

**Intervention**. Participants received KME supplements (DeltaG Tactical Ketones, TdeltaS Global, Inc.) twice daily based on stratified weight-based dosing. The first dose was post-breakfast: light weight <72.5 kg receiving 27 g KME; medium weight 72.5 kg–90.7 kg receiving 35 g KME; and heavy weight >90.7 kg receiving 42 g KME. The second dose was 1.5h after the first dose: light weight 9 g KME; medium weight 11.7 g KME; and heavy weight 14 g KME.

This two-stage dosing protocol was designed to emulate a field-compatible supplementation routine relevant to tactical and operational environments, emphasizing practicality and sustained ketone exposure. The goal was to achieve and sustain ketosis, with concentrations comparable to those observed under nutritional ketosis or intermittent fasting, for approximately 4–5 h.

**Measurement**. Below are the list of devices and instruments used for data collection and their descriptions:

**Abbott Keto Lingo continuous ketone monitor (CKM)**. The Abbott Keto Lingo CKM is a continuous monitoring device designed to measure ISF BHB levels every 5 min [12]. It uses an ultra-thin needle sensor inserted subcutaneously, which continuously tracks ketone concentration and wirelessly transmits data to a beta version of a proprietary mobile application provided by Abbott Lingo.
**Abbott Freestyle Libre2 continuous glucose monitor (CGM)**. The Abbott FreeStyle Libre 2 CGM is a widely used device for real-time monitoring of ISF glucose levels [18]. The sensor measures glucose once per minute and stores 15-min averaged readings in its onboard memory for up to 8 h before upload. Similar to the CKM, it uses an ultra-thin needle sensor inserted subcutaneously, continuously tracking glucose concentration and wirelessly transmitting data to an accompanying mobile phone application.
**Sensor insertion and placement**. Continuous ketone monitoring (CKM) and continuous glucose monitoring (CGM) sensors were applied by trained study personnel following the manufacturers' guidelines. Each sensor contained a microneedle array designed to sample interstitial fluid (ISF) at a consistent depth within the dermis. For all participants, sensors were placed on the volar aspect of the upper arm, approximately 5 cm above the elbow crease, avoiding visible veins or scars. This site was selected because it offers low mechanical stress, minimal muscle movement, and consistent subcutaneous thickness across individuals, thereby reducing variability in ISF kinetics. The microneedle was inserted perpendicularly to the skin surface using the manufacturer-provided applicator, which ensures a fixed insertion force and depth. The area was cleaned with isopropyl alcohol, allowed to dry, and the skin was gently flattened during application to standardize insertion angle. Sensors were held in place with medical-grade adhesive and covered with transparent film to minimize mechanical displacement. Following insertion, participants remained seated for approximately 10 min to allow local equilibration before recording began. Any insertion attempts with visible bleeding or abnormal resistance were discarded and replaced. The microneedle-based ISF sampling approach used in the current study is well-validated as a minimally invasive and reliable technique for continuous biochemical monitoring in humans, with demonstrated reproducibility across participants when standardized by site and insertion depth [19,20].
**Oura Ring (Gen 3)**. The Oura Ring Gen 3 is a lightweight, multi-sensory wearable designed for monitoring activity, recovery, and sleep metrics [17,21]. Worn as a ring, the device captures beat-by-beat blood volume pulse data using infrared light and a photosensor positioned over the palmar artery of the ring finger. An integrated 3D accelerometer continuously records body movement amplitude and intensity with 30-s frequency resolution, providing fine-grained actigraphy-based estimates of activity and sleep. Additionally, a negative temperature coefficient thermistor measures finger skin temperature every 60 s with a precision of .07 °C, allowing for detailed tracking of nocturnal temperature variations. Sleep–wake patterns, as well as heart rate/heart rate variability (HR/HRV) characteristics, are derived from a combination of pulse rate dynamics, beat-to-beat interval variability, pulse amplitude changes, and motion features, integrating multiple physiological signals to provide a comprehensive sleep profile. We used Oura's sleep and readiness metrics to assess KME supplementation effect on sleep (See Table 2 for the list of metrics and definitions).
**Automated Self-Administered 24-h dietary assessment tool (ASA24)**. The ASA24 is a validated and widely utilized online platform developed to capture detailed dietary intake data. The ASA24 enables analysis of nutritional factors in health outcomes and serves as an instrument for quantifying participants' dietary intake, supporting the evaluation of metabolic responses to interventions [22]. It facilitates self-reporting of food and beverage consumption over 24 h, guiding users through meal recall with prompts and visual aids to enhance accuracy and completeness. The tool incorporates standardized food databases to estimate energy, macronutrient, and micronutrient intake. Participants filled out ASA24 daily. We used daily calorie intake and macro nutrients balance to characterize participants' metabolic state in relation to KME response and other data streams.
**Dual-Energy X-ray Absorptiometry (DXA) Scan**. Total body scans were conducted on Day 1 via a GE Lunar iDXA (GE Healthcare, Chicago, IL, USA) and analyzed using enCore Version 18 software to provide total body and partitioned results for bone, fat and lean tissue compartments. The scan was conducted by a trained technician according to the manufacturer's recommendations. We used DXA to assess body composition, including bone mineral density (BMD), bone mineral content (BMC), lean mass, and fat mass.
**Blood biomarkers**. Fasted venous blood samples were obtained on Days 1 and 14 for complete blood count with differential (CBC) and comprehensive metabolic panel (CMP) analysis which were performed by Quest Diagnostics (Quest Diagnostics, Tampa, FL). We used CBC and CMP panels to assess hematologic and metabolic effects of 10 days of KME supplementation.

**Procedure**. The procedure during the 14-day study is described below:

**Day 0: Orientation**. Participants attended an in-person orientation session where they provided informed consent and received instructions regarding the study protocol. During this session, participants were familiarized with both the devices and their corresponding applications, including the Abbott Keto Lingo CKM beta app, Abbott Freestyle Libre 2 CGM app, and the Oura app. Study staff demonstrated device setup, Bluetooth pairing, data synchronization, and troubleshooting procedures, and participants practiced each step under supervision until confident in their use. They were also trained to log their dietary intake using the ASA24. Automated daily reminders for participants were set up to aid with study compliance.
**Days 1**–**4: Baseline Phase**. During the baseline phase, participants were monitored during normal diet and activity without intervention. On the first day of this phase, participants' weight and body composition were assessed using DXA. CGM and CKM were applied. Participants were instructed to begin daily logging of dietary intake via ASA24 and to continuously wear the Oura Ring to track physical activity and sleep. Participants synced their devices daily and provided reports to the study coordinator regarding device use, and deviation of their diet and physical activity from normal. These data were used to establish a pre-intervention baseline.
**Days 5**–**14: Intervention Phase**. The intervention phase included 10 days of KME supplementation administered twice daily. KME supplement intake was remotely monitored via a video call with a research team member. Participants were instructed to maintain their normal diet and activity. Participants reported dietary intake, physical activity, and any KME adverse effects daily. CGM and CKM data were collected continuously. Participants synced wearable devices at the end of each day.
**Day 14: Final Assessment**. On the final day, weight, BMI, and DXA body composition were reassessed. A fasted blood draw was performed. Participants consumed breakfast and their final KME doses under observation. Later that day, CGM and CKM devices were removed and device-related mobile applications were uninstalled.

**Statistics**. All analyses were conducted using R (version 4.5.1) and Python (version 3.13.7). Descriptive statistics are presented as mean ± standard deviation unless otherwise noted. Primary pharmacokinetic parameters for interstitial-fluid β-hydroxybutyrate (BHB) were derived for each participant using custom scripts that identified individual peak amplitude, time-to-peak, and decay half-life based on fitted dose-response curves. Within-subject comparisons across baseline and supplementation phases were evaluated using paired t-tests.

Granger causality analyses were applied to time-series data from CKM and CGM to examine temporal relationships between BHB and glucose signals, using 1-min sampling intervals and a 5-min lag window to identify directional effects.

Correlational analyses explored associations among KME pharmacokinetic variables, body composition (DXA), dietary intake (ASA24), and behavioral–metabolic indices (Oura and CGM metrics). Pearson's product-moment correlation coefficients (r) were computed for all variable pairs. Given the modest sample size (n = 16) and the exploratory objective of identifying potential predictors of ketone absorption and clearance, correlational analyses were evaluated at a relaxed alpha threshold of p < .10. This approach is consistent with prior small-sample feasibility studies aimed at identifying candidate relationships for hypothesis generation rather than confirmatory inference.

## 2 Results

For the primary analysis of ISF BFB levels, we excluded data based on CKM data availability and normality at the participant and individual day levels. Specifically, a participant's data was excluded if more than 80 % of the total data were missing. This threshold was determined post-hoc after visual inspection of data completeness to ensure that included participants contributed sufficient valid data for reliable curve fitting. We also removed data from a participant with abnormal CKM readings due to nutritional ketosis or inaccurate sensor readings. At the individual day level, we excluded a given day if more than 50 % of the data were missing or if the peak of the CKM dose response curve was missing. In total, this data exclusion process resulted in removal of 4 participants and 10 % of total monitoring days from the remaining 16 participants.

Most excluded days reflected intermittent Bluetooth connectivity interruptions that prevented data synchronization during free-living use. These lapses occurred sporadically across both baseline and intervention phases and were not associated with systematic time effects. Because participants were monitored in naturalistic conditions without remote telemetry access, data quality could only be verified after device retrieval at the end of the study. Importantly, exclusions from CKM analyses were due to device malfunction or excessive data loss rather than participant noncompliance. All participants adhered to the KME supplementation protocol which was confirmed during daily video call proctoring. Therefore, all 20 participants were included in the analysis not related to CKM, such as the examination of the potential effects of KME supplementation on sleep metrics and blood biomarkers.

**Fig. 1:**
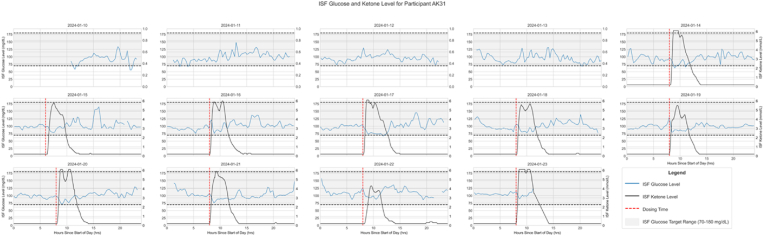
One participant's ISF BHB (black line) and glucose (blue line) levels plotted over 24 h for each day of the 14-day participation.

We interpolated missing data in the individual day data that passed the above exclusion criteria. Linear interpolation was used to fill any missing data within the dose-response time window. The start of this window was defined as when the KME dose was taken, and the ending time was defined as the dose time plus the max duration of elevated ketone blood ketone levels specific to each participant. Missing data outside of the dose response time was set to a baseline level of .2 mmol/L. Fig. 1 shows one representative participant's ISF glucose and ketone level plotted over 24 h for each day of the 14-day participation. The same figures for the remaining participants are reported in Supplementary Materials (Fig. S1).

### 2.1 Pharmacokinetics of KME supplementation

**Fig. 2:**
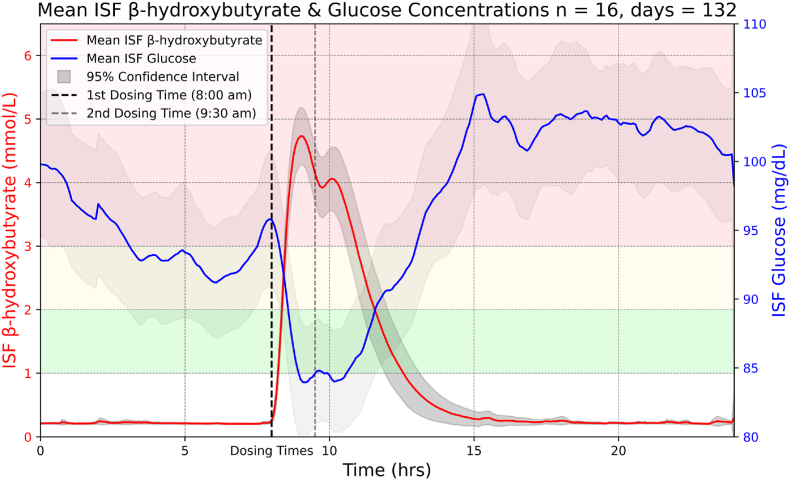
ISF BHB and glucose dose-response curves from weight stratified KME dosing averaged across available participants and days.

We extracted several key absorption and clearance metrics to characterize BHB pharmacokinetics resulting from KME supplementation: Tmax, Cmax, onset_time, offset_time, AUC, and half_life. The definitions along with mean and SD are provided in Table 1. Fig. 2 shows the BHB dose-response curve of KME supplementation along with the ISF glucose level averaged across all available participants and days.

**Table 1:** The definitions and descriptive statistics of the KME Pharmacokinetics metrics.

Feature	Unit	Definition	*M*(*SD*)
Tmax	hrs	Time to peak concentration, Time at which the compound reaches its maximum concentration in the system	1.17(.63)
Cmax	mmol/L	Peak concentration, highest observed concentration of the compound	5.27(.93)
onset_time	hrs	Time when the compound first reaches .5 mmol/L (start time = dosing time)	.24(.23)
offset_time	hrs	Time when the compound is no longer above .5 mmol/L (start time = dosing time)	5.12(1.31)
AUC	hrs ∗ mmol/L	Total drug exposure over time, representing area under the concentration curve (only calculated area when ckm ≥ .5 mmol/L, .5 is treated as the floor when calculating area)	12.11(4.69)
half_life	hrs	Time required for the compounds concentration to decrease by half	1.97(.76)
glucose_supression_magnitude	mg/dL	The drop in glucose level from the time the ketone level peaked (Cmax) down to a glucose level minimum	22.09(10.43)
glucose_suppression_time	hours	The time taken to reach a minimum glucose level starting when the ketone level peaks (Cmax)	1.81(.94)

**Table 2:** The definitions and descriptive statistics of sleep, activity, body composition, demographics, CGM, and diet metrics.

Data Stream	Feature	Unit	Definition	M(SD)
**OURA**	sleep_score	Range 0-100	Overall measure of how well you slept, reflecting nightly sleep duration, efficiency, latency, and timing (higher = better)	71.83(5.65)
	hrv_average	ms	Constant variation in milliseconds between your heartbeats is known as your heart rate variability	58.69(18.25)
	readiness_score	Range 0-100	Measures physiological recovery and preparedness for daily activity, reflecting HRV, resting heart rate, temperature deviation, and prior sleep history (higher = better)	73.16(4.77)
	daily_movement	meters	Distance traveled in a day	8076.37(3805.80)
	steps	steps	Number of steps taken in a day	7910.10(2738.06)
	inactive	hours	Time spent in very low movement or resting	8.86(1.29)
	activity_score	Range 0-100	Overall measure of your activity, training, and recovery balance	80.96(13.62)
	avg_resting_hr	bpm	Average resting heart rate when sleeping	60.78(7.09)
**Daily Questionnaire**	exercise_frequency	days/week	Number of days per week participants exercised	3.01(1.46)
**DXA & Demographics**	Weight	kg	The participant's total body weight	74.58(18.76)
	Height	cm	The participant's height	170.32(8.07)
	Bone Mass	g	The total estimated bone mass	2666.88(406.49)
	Fat Mass	g	The total estimated fat mass	20428.44(7926.06)
	Lean Mass	g	The total estimated lean mass in the body, excluding fat and bone	47856.18(8998.78)
	Tissue_pfat	%	The percentage of fat relative to soft tissue mass, excluding bones	29.53(7.81)
	Tissue Area	cm^2^	The total scanned area of soft tissue	5546.98(572.90)
	BMD	g/cm^2^	Bone Mineral Density, representing bone strength; higher values indicate better bone health	1.18(.13)
	BMC	g	Bone Mineral Content, the total amount of mineral content in bones	631.16(106.13)
	Bone Scan Area	cm^2^	The total area of the body that was scanned during DXA assessment	529.92(50.61)
	Age	years	The participant's age calculated from their birth year	28.44(4.34)
**CGM**	Average Glucose	mg/dL	The mean glucose level recorded by the CGM over the monitoring period	95.88(9.74)
	Glucose Management Indicator (GMI)	%	A predicted HbA1c estimate derived from CGM data	5.59(.22)
	Glucose Variability	%	The coefficient of variation (CV) of glucose levels, indicating fluctuations; higher values mean greater variability	15.57(3.14)
	Target Glucose Range	%	The percentage of CGM readings within the optimal glucose range (70–180 mg/dL) considered ideal for maintaining glucose stability	93.75(13.88)
**ASA24**	kcal	kcal	Average daily calorie intake	1970.05(567.27)
	prot	g	Average daily protein intake	92.56(27.33)
	tfat	g	Average daily trans fat intake	86.42(32.33)
	carb	g	Average daily carbohydrate intake	195.16(52.41)
	prot_kcal_ratio	%	Average daily protein intake in proportion to total calorie intake	.19(.03)
	tfat_kcal_ratio	%	Average daily trans fat intake in proportion to total calorie intake	.39(.07)
	carb_kcal_ratio	%	Average daily carbohydrate intake in proportion to total calorie intake	.40(.06)

The time to peak concentration (Tmax = 1.17 ± .63 h) suggests that KME is rapidly absorbed into circulation following ingestion, with peak BHB levels occurring within approximately 1 h. This fast absorption is likely due to the efficient hydrolysis of KME by intestinal and plasma esterases, producing BHB and 1,3-butanediol. The latter is further metabolized in the liver to generate additional BHB, contributing to a sustained elevation in circulating ketones [10].

The peak concentration (Cmax = 5.27 ± .93 mmol/L) indicates a substantial rise in circulating ketones, well above physiological fasting levels (typically .2–.5 mmol/L) and comparable to the upper range of ketosis observed during prolonged fasting or ketogenic diets. This suggests that KME effectively induces a hyperketotic state, which may be beneficial for applications requiring rapid ketone availability when ketogenic diet is not feasible.

The onset of ketosis (onset_time = .24 ± .23 h) confirms that BHB rises almost immediately following KME ingestion, reaching a threshold of .5 mmol/L within 15 min. This rapid onset suggests KME's potential as an acute metabolic intervention, particularly in scenarios where rapid ketone availability is desired.

Conversely, the offset time (offset_time = 5.12 ± 1.31 h) after initial and booster doses suggests that the ketone elevation persists for approximately 5 h before returning to baseline levels. This relatively prolonged elevation compared to ketone salts or medium-chain triglycerides (MCTs) suggests that KME provides a sustained ketogenic state, perhaps due to slower hepatic metabolism and clearance of BHB.

The area under the curve (AUC = 12.11 ± 4.69 h∗mmol/L) quantifies total ketone exposure, reflecting both the peak and duration of ketosis. The moderate variability in AUC values suggests inter-individual differences in ketone metabolism, potentially influenced by factors such as diet, exercise, baseline metabolic state, liver function, and previous exposure to ketones.

The half-life (t_1_/_2_ = 1.97 ± .76 h) is consistent with the expected metabolic clearance of ketones, suggesting that once absorbed, BHB is rapidly utilized by tissues or cleared via renal excretion. The half-life supports KME's sustained effects, distinguishing it from shorter-lived ketone supplements such as ketone salts.

The glucose-suppression magnitude (22.09 ± 10.43 mg/dL) shows that circulating glucose fell by roughly one-fifth of a typical baseline level after KME ingestion, confirming a meaningful but transient hypoglycemic effect. The sizeable spread suggests inter-individual differences potentially related to baseline insulin sensitivity and hepatic glycogen status.

The glucose-suppression time (1.81 ± .94 h) reveals the local lowest glucose level occurred on average about 2 h after the ketone peak, consistent with the expected delay between maximal BHB level and downstream metabolic effects. The wide range suggests variability in hepatic and peripheral glucose handling across participants.

**Fig. 3:**
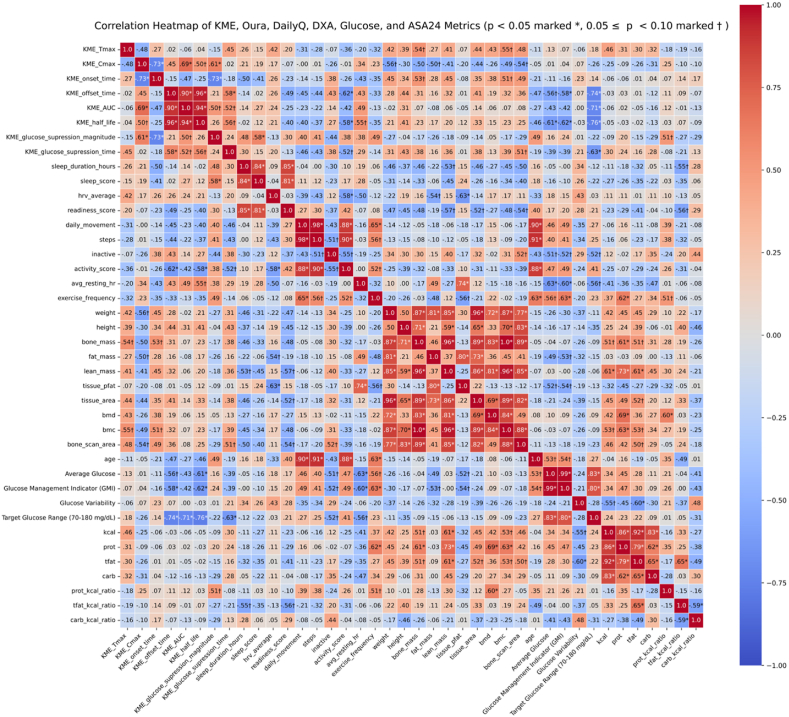
Color-coded correlation matrix of KME pharmacokinetics features, CGM features, DXA and demographic variables, and ASA24 variables.

Next, we validated these and metrics from other data streams relevant to metabolism (CGM, ASA24, daily questionnaire, and DXA scan/demographics) by running a correlational analysis using Pearson's product moment correlation. The list of metrics from each data stream that were included in the analysis is shown in Table 2 and the correlation matrix including all these variables are shown in Fig. 3.

### 2.2 Internal correlation among KME supplementation pharmacokinetic features

Eight KME pharmacokinetic features formed a highly coherent profile, including 12 pairwise associations meeting the *p* < .10 criterion. A shorter onset of ketosis was linked to a higher peak BHB concentration (*C*max vs. onset_time: *r* = −.73, *p* = .007). Greater *C*max coincided with a larger total ketone exposure (*AUC*: *r* = .69, *p* = .013) and a longer apparent half_life (*r* = .50, *p* = .099). The features indexing elimination, such as offset_time, *AUC*, and half_life were tightly coupled (*offset_time vs. AUC*: *r* = .90, *p* < .001; *offset_time vs. half_life*: *r* = .96, *p* < .001; *AUC vs. half_life*: *r* = .94, *p* < .001). Larger peaks and faster onsets predicted greater glucose_suppression_magnitude (*C*max: *r* = .61, *p* = .037; onset_time: *r* = −.73, *p* = .007). Longer offset_time and longer half_life tracked with a prolonged glucose suppression duration (*r* = .58-.56, *p* ≤ .06). Total exposure (*AUC*) related positively to both suppression magnitude (*r* = .50, *p* = .096) and duration (*r* = .52, *p* = .085).

### 2.3 Correlation between KME pharmacokinetic features and other data streams

Several body composition, lifestyle, and glycemic control metrics showed modest associations with the eight KME pharmacokinetic/glucose suppression features (all *p* < .10). *KME uptake features* correlated positively with DXA derived bone mass and bone mineral content (*T*max vs bone_mass: *r* = .54, *p* = .068; *T*max vs bmc: *r* = .55, *p* = .066; *onset_time* vs bone_mass: *r* = .53, *p* = .08; onset_time vs bmc: *r* = .52, *p* = .09), indicating that participants with larger bones reached peak ketone levels more slowly. Peak BHB concentration (*C*max) showed inverse relationships with bodyweight (*r* = −.56, *p* = .061), total fat mass (*r* = −.50, *p* = .095) and bone scan area (*r* = −.54, *p* = .072), suggesting a dilutional or distribution volume effect in heavier or more adipose individuals. Lower habitual activity predicted slower ketone clearance (offset_time: *r* = −.62, *p* = .030; half_life: *r* = −.58, *p* = .046). Half_life also tended to rise with higher resting heartrate (*r* = .55, *p* = .064). Better day-to-day glycemic control, reflected in lower average glucose and lower GMI was associated with a longer elimination phase (average glucose vs offset_time: *r* = −.56, *p* = .058; average glucose vs half-life: *r* = −.61, *p* = .035; GMI vs offset_time: *r* = −.58, *p* = .049; GMI vs half-life: *r* = −.62, *p* = .031). This pattern was robust for the percentage of CGM readings within the target glucose range (70–180 mg/dL) (Target Glucose Range vs offset_time: *r* = −.74, *p* = .006; Target Glucose Range vs half_life: *r* = −.76, *p* = .004). Greater total ketone exposure (*AUC*) and longer glucose suppression time were both linked to spending less time within the target glucose range (*AUC*: *r* = −.71, *p* = .009; suppression time: *r* = −.63, *p* = .027). In addition, better sleep and higher protein intake were associated with the magnitude of glucose suppression (sleep_score vs glucose_suppression_magnitude: *r* = .58, *p* = .049; prot_kal_ratio vs glucose_suppression_magnitude: *r* = .51, *p* = .09) whereas lower habitual activity and larger bone scan area were associated with longer glucose suppression time (activity_score vs glucose_suppression_time: *r* = −.52, *p* = .08; bone_scan_area vs glucose_suppression_time: *r* = .51, *p* = .09). Taken together, these findings indicate that skeletal and adipose mass modulate ketone onset and peak amplitude, whereas habitual activity and baseline glycemic health shape how long exogenous ketones remain elevated and how long they suppress glucose.

While we elected to report only the correlations including the KME pharmacokinetics features in this section for brevity, the complete list of correlations that had p > .10 is reported in Supplemental Material (Table S1).

### 2.4 Granger causality analysis of ketone-induced glucose suppression

KME supplementation has a known effect of glucose level suppression [10]. This is also evident in Fig. 2 which shows a double peak in ISF ketone level, reflecting the initial dose followed by the booster, and a corresponding double dip in glucose level. To examine the temporal relationship between ISF ketone and glucose levels following KME ingestion, we conducted a Granger causality analysis. This method determines whether past values of the predictor variable (BHB) significantly improve the prediction of the outcome variable (glucose) beyond what glucose levels alone can predict. Prior to analysis, the dataset was filtered to include only time points corresponding to KME ingestion and the subsequent 7-h post-dose window. We visually inspected the individual participant-day plots (see Fig. S1) and excluded the daily data points that did not provide sufficient data. ISF glucose data, which were originally sampled every 15 min, were linearly interpolated to match the 5-min resolution of ISF BHB measurements, ensuring alignment in the time series. The Granger causality test was performed using a Vector Autoregression (VAR) model, with lags ranging from 5 to 60 min (in 5-min increments). The VAR model was defined as:

$$G_{t}=\alpha_{0}+\sum_{i=1}^{p}\alpha_{i}G_{t-i}+\sum_{i=1}^{p}\beta_{i}K_{t-i}+\epsilon_{G,t}$$

$$K_{t}=\gamma_{0}+\sum_{i=1}^{p}\gamma_{i}K_{t-i}+\sum_{i=1}^{p}\delta_{i}G_{t-i}+\varepsilon_{K,t}$$

where *G*_*t*_ represents ISF glucose at time *t*, *K*_*t*_ represents ISF BHB at time *t*, *p* is the lag order (ranging from 5 to 60 min), and ϵ represents the error terms. The null hypothesis for Granger causality testing was:

$$H_{0}:\beta_{1}=\beta_{2}=\ldots =\beta_{p}=0$$

which states that past values of ketones do not significantly improve the prediction of glucose. Statistical significance was assessed using F-tests, with a threshold of p < .05. The p-values for each tested lag were visualized to identify time windows where BHB significantly predict future glucose levels. The analysis was conducted using Python (version 3.14) with the statsmodels package [23] for time-series modeling. The VAR model was fitted using the default settings in statsmodels, with Akaike Information Criterion (AIC) used for lag selection.

**Fig. 4:**
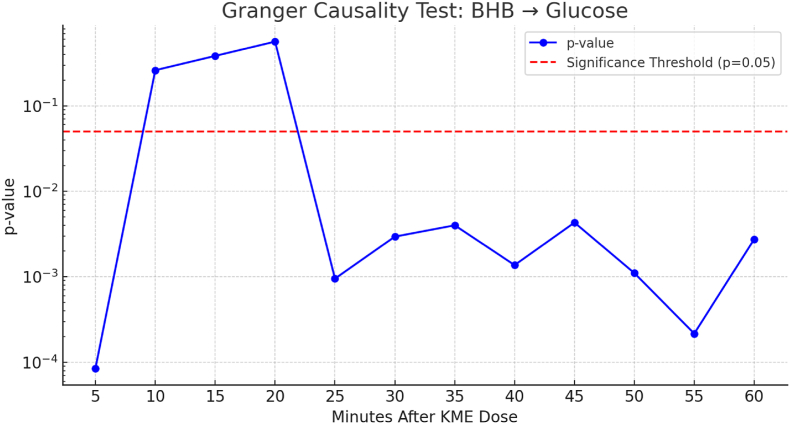
Granger causality analysis of ISF BHB levels predicting future ISF glucose levels following KME ingestion. The x-axis represents time lag (minutes after KME dose), while the y-axis shows the corresponding p-values from the Granger causality test. The red dashed line indicates the significance threshold (p = .05).

Fig. 4 shows the results of the Granger causality analysis, illustrating the temporal relationship between ISF BHB and glucose levels following KME ingestion. The Granger causality test revealed a significant predictive relationship between ISF BHB and glucose levels, characterized by three distinct phases: immediate effect (5 min post-KME), temporary plateau (10–20 min post-KME), and main suppression window (25–55 min post-KME). First, a highly significant effect was observed at 5 min post-dose (p < .0001), indicating an immediate influence of BHB on glucose suppression. This suggests that BHB exerts a near-instantaneous metabolic effect on glucose regulation. Between 10 and 20 min, the p-values rose above the significance threshold (p > .05), indicating a brief period where BHB levels did not significantly predict glucose fluctuations. This suggests a temporary stabilization phase where glucose suppression is not strongly driven by BHB. A second wave of highly significant Granger causality effects emerged between 25 and 55 min post-dose, with p-values dropping well below .05. This period represents the primary window where BHB exerts a sustained glucose-lowering effect. The results are consistent with known metabolic kinetics, as BHB metabolism typically peaks within 30–60 min post-ingestion, aligning with this observed effect.

**Fig. 5:**
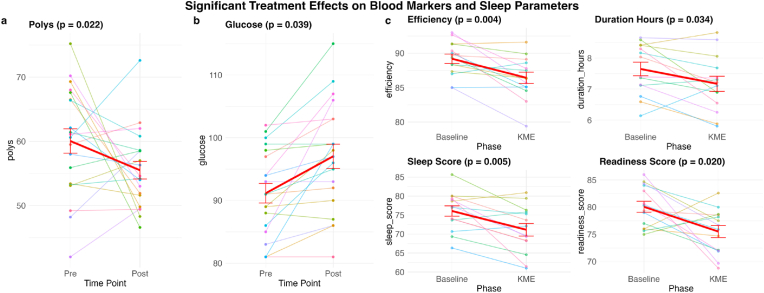
Pre-post or Baseline-KME phase plots along with the p-values of the paired-sample *t*-test for all variables that showed significant effects from - **a**: Complete Blood Count (CBC); **b**: Comprehensive Metabolic Panel (CMP); and **c**: Oura sleep metrics. The red line shows the group mean and other lines show individual participant's data. The error bars denote the standard error of the mean.

**Blood biomarkers**. We assessed the changes in blood biomarkers by running paired-sample t-tests comparing pre (Day 1) and post (Day 14) 10 days of KME supplementation for each variable in the CBC and CMP. In CBC, neutrophil percentage of all detected white blood cells (polys) was the only variable that showed significant pre-post change (p = .02: Fig. 5 a). The range of data observed (43–75 %) was within a normal range. This indicates that short-term KME supplementation did not significantly alter hematological parameters or function.

Of the CMP variables, glucose was the only variable that showed significant pre-post change. Specifically, blood glucose level increased post 10-day KME supplementation (p = .04: Fig. 5 b). Importantly, CMP variables indicative of liver function (bilirubin_total, alk_phos: alkaline phosphatase, ast: aspartate aminotransferase, and alt: alanine aminotransferase) did not show significant pre-post difference.

**Oura sleep metrics**. We examined the effects of KME supplementation on sleep by running paired-sample t-tests comparing the average of eight key variables from Oura in the Baseline and KME Intervention Phases. Sleep efficiency (p = .004), sleep duration (p = .034), sleep score (p = .005), and readiness-score (p = .020) showed significant reduction during the KME intervention Phase compared with the Baseline Phase (Fig. 5 c).

While we elected to show plots for only the variables that showed significant effects for brevity in this section, the figures depicting the changes and p-values for all variables in CBC (Fig. S2), CMP (Fig. S3), and Oura sleep metrics (Fig. S4) are reported in the Supplemental Materials.

## 3 Discussion

We conducted an ecologically valid observational study of 10-day KME supplementation under normal activity and diet using simultaneous CKM and CGM. To our knowledge, this is the first study to integrate dual analyte monitoring under free-living conditions, extending prior laboratory-based ketone kinetics research [10,24] into an applied context.

This approach enabled the continuous capture of ketone–glucose dynamics and their broader physiologic correlates in real-world settings, complementing prior work using controlled dosing and intermittent blood sampling [25]. The results demonstrated that KME supplementation leads to a rapid increase in circulating BHB, with peak levels occurring within an hour and ketosis persisting for approximately 5 h, consistent with previous findings from exogenous ketone studies [10,26].

However, two important caveats should be considered when interpreting these findings. First, the CGM used in this study was limited to a maximum reading of 6.0 mmol/L, which may have underestimated some Cmax values, particularly in individuals who reached or exceeded this threshold. Examination of individual daily plots (Fig. S1) confirmed that a few participants consistently reached the upper bound of this reading range. As a result, the true peak concentrations (Cmax) may be underestimated, and the variability in ketone exposure (AUC) could be artificially constrained. Second, these pharmacokinetic parameters were derived from a protocol incorporating weight stratification and booster dosing, wherein participants received approximately 415–442 mg/kg KME initially (27 g, 35 g, 42 g KME for light <72.5, medium 72.5 kg–90.7 kg, and heavy >90.7 kg respectively), followed by a 1/3 booster dose 1.5 h later—an approach adapted from prior work optimizing sustained ketosis for performance and operational tolerance [27,28]. This dosing strategy extended the observed ketone elevation, influencing both the offset time and total ketone exposure. This dosing protocol was developed for use in operational settings and balances metabolic effects and logistical feasibility. Prolonged ketosis has been linked to enhanced cellular energy efficiency [29] and may promote resilience to metabolic or oxidative stress, including after neural injury or energy-deficit states [[30], [31], [32]].

Exploratory correlational analysis suggested a two-stage control architecture governing exogenous ketone dynamics. Preliminary trend indicated that skeletal and adipose mass (bone mass, fat mass, tissue area) tended to delay ketone uptake and blunted peaks concentrations, whereas behavioral-metabolic factors, such as habitual activity, sleep quality, protein intake, and baseline glycemic health, appeared to influence how long exogenous ketones stayed elevated and how long they suppressed glucose. These results align with prior reports linking higher lean mass to faster ketone clearance [25] and habitual exercise to enhanced metabolic flexibility [33,34]. Although these associations should be interpreted as hypothesis-generating given the small sample size, they collectively suggest that body composition sets the “ketone capacity,” while lifestyle modulates “ketone clearance,” and these inform precision-dosing frameworks.

Granger causality analysis confirmed the causal role KME supplementation takes in suppressing ISF glucose level and determined the time course of this effect. Specifically, BHB levels significantly predicted future glucose suppression, with two key periods of effect: an immediate response at 5 min and a stronger suppression phase between 25 and 55 min post-dose. These findings, enabled by the simultaneous use of CKM and CGM, extend prior evidence that exogenous ketones acutely reduce hepatic glucose output and enhance peripheral glucose uptake [7,10].This temporal specificity highlights the potential for precisely timed KME administration to modulate glycemic control, which could be particularly relevant for optimizing metabolic interventions in clinical populations such as individuals with diabetes, or in athletes seeking to enhance metabolic flexibility for performance and recovery.

Clinical laboratory analysis indicated that 10 days of KME supplementation led to significant elevation in fasting blood glucose levels. An interesting finding in this analysis was that the fasted blood glucose level increased after 10 days of KME supplementation. This is surprising given the KME's acute glucose suppression effect that was observed in the current study and several others [7,10]. The absence of significant changes in liver function markers suggests that KME is well tolerated from a hepatic perspective. Potential mechanisms for this effect include suppression of hepatic glucose production via reduced free fatty acid (FFA) availability and gluconeogenic inhibition [10], ketones becoming body's preferred energy substrate [35], and altering hormonal signaling (e.g., glucagon) [27]. We suggest that the elevated glucose level after the 10-day KME supplementation emerged as a result of a compensatory adaptation in response to these mechanisms underlying the acute glucose suppression effect.

A few sleep metrics (sleep efficiency, sleep duration, sleep score, and readiness-score) showed statistically significant decline during the KME intervention phase compared to the Baseline phase. This finding needs to be interpreted with caution due to the small sample size and a lack of control in our study design. Prior research suggests that ketone bodies impact circadian rhythm, and exogenous ketone supplementation improves sleep quality in healthy adults as well as improves sleep efficiency after strenuous exercise[36]; [37,38]. Our data suggest that multiple days of continuous KME supplementation could impact sleep, and additional research is needed to discern if ingestion of exogenous ketone bodies in the morning KME alters circadian rhythm and metabolic entrainment.

**Limitations**. Several limitations should be acknowledged. First, although sensor insertion and anatomical site were standardized, small individual differences in skin thickness, subcutaneous fat, and hydration may have introduced minor variability in interstitial fluid kinetics. Second, dietary intake was not standardized; therefore, day-to-day variability in macronutrient composition and meal timing could have influenced glycemic dynamics. Third, approximately 10 % of monitoring days were excluded due to sensor detachment or signal loss, and four participants were removed based on pre-specified compliance criteria. Future work should incorporate redundant sensors or enhanced participant training to further minimize data loss. Fourth, the modest sample size limits the power of correlational analyses; therefore, relationships between metabolic and behavioral variables should be interpreted as exploratory and hypothesis-generating. Fifth, the CKM device had an upper detection limit of approximately 6 mM β-hydroxybutyrate (BHB), and several participants occasionally reached or briefly exceeded this threshold. While the dosing protocol was designed to elicit moderate, sustained ketosis within the device's measurable range, ceiling effects may have led to slight underestimation of true peak BHB levels. Future studies using higher KME doses or examining inter-individual variability should employ devices capable of quantifying beyond this range to more fully capture the kinetics of exogenous ketosis. Finally, the observed increases in fasting glucose and transient changes in sleep quality warrant further mechanistic investigation to determine whether they represent adaptive metabolic adjustments or transient perturbations associated with sustained exogenous ketone exposure.

## 4 Conclusion

This study provides ecologically valid insights into BHB pharmacokinetics of KME supplementation and its downstream effects on glucose regulation and sleep. Our findings demonstrate that a weight stratified repeated dose KME supplementation protocol leads to a rapid rise in ISF BHB with peak levels occurring within an hour and elevation lasting for approximately 5 h. Exploratory analyses suggested that ketone absorption and clearance were strongly modulated by dietary energy intake, body-composition indices, habitual physical activity, and baseline glycemic control, highlighting potential avenue for individualized dosing. Despite acute glucose suppression, fasting glucose rose modestly after 10 days of high-dose KME supplementation, suggesting possible compensatory metabolic adaptation. This finding underscores the complexity of ketone-glucose interactions and highlights the need for further research into the long-term metabolic effects of chronic ketone supplement use.

Additionally, the study revealed a potential impact of KME supplementation on sleep, with reductions in sleep efficiency, duration, and overall sleep quality metrics whereas HRV remained stable. Given the small sample size, observational design, and exploratory statistical threshold (p < .10), these findings should be interpreted as preliminary trends rather than confirmatory effects. They nonetheless raise important questions regarding the physiological consequences of prolonged exogenous ketone intake on circadian and neuroendocrine regulation.

Overall, this study demonstrates the feasibility and scientific value of simultaneous continuous ketone and glucose monitoring (CKM and CGM) for capturing the dynamic metabolic effects of exogenous ketone supplementation. The findings provide initial framework for understanding the balance between the acute benefits and potential compensatory responses associated with KME supplementation, emphasizing the importance of wearable analyte monitors for personalized health and performance applications. Future studies should validate and investigate the mechanistic underpinnings of these exploratory findings in larger, more controlled settings to optimize ketone-based interventions for metabolic health and performance.

## CRediT authorship contribution statement

**Toshiya Miyatsu:** Conceptualization, Formal analysis, Methodology, Supervision, Validation, Visualization, Writing – original draft, Writing – review & editing. **Connor Tate:** Conceptualization, Data curation, Investigation, Methodology, Project administration, Writing – review & editing. **Jeremy McAdam:** Conceptualization, Data curation, Investigation, Methodology, Project administration, Writing – review & editing. **Chandler Massey:** Formal analysis, Visualization, Writing – original draft. **Timothy Broderick:** Conceptualization, Methodology, Project administration, Supervision, Writing – review & editing.

## Declaration of generative AI and AI-assisted technologies in the manuscript preparation process

During the preparation of this work the authors used Perplexity, Claude, and ChatGPT in order to assist with literature search and to identify and correct typographical and grammatical errors. After using this tool/service, the authors reviewed and edited the content as needed and take full responsibility for the content of the published article.

## Conflict of interest

Abbott Lingo provided continuous ketone and glucose monitoring devices as in-kind support for this study. The authors received no financial compensation from Abbott and declare that they have no other known competing financial interests or personal relationships that could have appeared to influence the work reported in this paper.
