# Supplementary material for: Pharmacokinetics and metabolic effects of ketone monoester supplementation: The first simultaneous CKM and CGM study under normal diet and activities
Source: Metabol Open. 2025 Oct 29;28:100411. doi: 10.1016/j.metop.2025.100411 (PMC12617643; doi:10.1016/j.metop.2025.100411)
Supplement: Multimedia component 1 [file mmc1.docx]

STROBE Statement—Checklist of items that should be included in reports of ***cohort studies***

|  | Item No | Recommendation |
| --- | --- | --- |
| **Title and abstract** | 1 | (*a*) Indicate the study’s design with a commonly used term in the title or the abstract: **Yes** – “In this single-group observational study, twenty healthy adults underwent …” (Abstract). |
|  |  | (*b*) Provide in the abstract an informative and balanced summary of what was done and what was found: **Yes** – includes background, methods, results, and conclusions. |
| Introduction | | |
| Background/rationale | 2 | Explain the scientific background and rationale for the investigation being reported: **Yes** – exogenous ketone supplementation and knowledge gap in real-world pharmacokinetics have been explained. |
| Objectives | 3 | State specific objectives, including any prespecified hypotheses: **Yes** - Objectives clearly stated: characterize pharmacokinetics of KME under free-living conditions; examine glucose suppression; explore effects on sleep and biomarkers. |
| Methods | | |
| Study design | 4 | Present key elements of study design early in the paper: **Yes** – Design described early in Methods: observational, baseline (4 days) + intervention (10 days). |
| Setting | 5 | Describe the setting, locations, and relevant dates, including periods of recruitment, exposure, follow-up, and data collection: **Yes** – “The recruitment and data collection were conducted at XXX(the institution’s name has been removed)XXX and remotely from January 10 to 25, 2024.” (Participants) |
| Participants | 6 | (*a*) Give the eligibility criteria, and the sources and methods of selection of participants. Describe methods of follow-up: **Yes** – Specified in Participants. |
|  |  | (*b*) For matched studies, give matching criteria and number of exposed and unexposed: **N/A** |
| Variables | 7 | Clearly define all outcomes, exposures, predictors, potential confounders, and effect modifiers. Give diagnostic criteria, if applicable: **Yes** – Specified in Methods. |
| Data sources/ measurement | 8* | For each variable of interest, give sources of data and details of methods of assessment (measurement). Describe comparability of assessment methods if there is more than one group: **Yes** – Specified in Methods. |
| Bias | 9 | Describe any efforts to address potential sources of bias: **Yes** - Potential biases noted: sensor accuracy limits, missing data; addressed by exclusion thresholds and interpolation rules (Methods – Results). |
| Study size | 10 | Explain how the study size was arrived at: **Yes** – “The sample size was based on a previous study of similar kind.” (Participants) |
| Quantitative variables | 11 | Explain how quantitative variables were handled in the analyses. If applicable, describe which groupings were chosen and why: **Yes** – Continuous variables analyzed; derived pharmacokinetic parameters defined (Tmax, Cmax, AUC, etc.) (Table 1). Categorization explained (e.g., weight stratification for dosing). |
| Statistical methods | 12 | (*a*) Describe all statistical methods, including those used to control for confounding: **Yes** - Statistical methods described: Pearson correlations, Granger causality analysis, paired t-tests. |
|  |  | (*b*) Describe any methods used to examine subgroups and interactions: **Yes** – Correlations between KME pharmacokinetic features and other data streams reported. |
|  |  | (*c*) Explain how missing data were addressed: **Yes** – exclusion thresholds + linear interpolation reported |
|  |  | (*d*) If applicable, explain how loss to follow-up was addressed: N/A |
|  |  | (*e*) Describe any sensitivity analyses: N/A |
| Results | | |
| Participants | 13* | (a) Report numbers of individuals at each stage of study—eg numbers potentially eligible, examined for eligibility, confirmed eligible, included in the study, completing follow-up, and analysed: **Yes** - Numbers at each stage reported: 20 recruited, 16 in CKM analysis |
|  |  | (b) Give reasons for non-participation at each stage: **Yes** – Reasons for non-participation/exclusion reported: poor/missing data |
|  |  | (c) Consider use of a flow diagram: **Yes** – we considered the use of a flow diagram but elected not use one based on the simple design of the study. |
| Descriptive data | 14* | (a) Give characteristics of study participants (eg demographic, clinical, social) and information on exposures and potential confounders: **Yes** – Table 2 |
|  |  | (b) Indicate number of participants with missing data for each variable of interest: **Yes** – the exclusion criteria and number of excluded participants reported. |
|  |  | (c) Summarise follow-up time (eg, average and total amount): N/A |
| Outcome data | 15* | Report numbers of outcome events or summary measures over time: **Yes** – all outcome measures reported. |
| Main results | 16 | (*a*) Give unadjusted estimates and, if applicable, confounder-adjusted estimates and their precision (eg, 95% confidence interval). Make clear which confounders were adjusted for and why they were included: **Yes** – means and SD or r and p-values reported |
|  |  | (*b*) Report category boundaries when continuous variables were categorized: **Yes** – categorization defined (Table 2) |
|  |  | (*c*) If relevant, consider translating estimates of relative risk into absolute risk for a meaningful time period: N/A |
| Other analyses | 17 | Report other analyses done—eg analyses of subgroups and interactions, and sensitivity analyses: **Yes** – detailed correlational analysis reported in Supplemental Materials |
| Discussion | | |
| Key results | 18 | Summarise key results with reference to study objectives: **Yes** – Key results summarized. |
| Limitations | 19 | Discuss limitations of the study, taking into account sources of potential bias or imprecision. Discuss both direction and magnitude of any potential bias: **Yes** – Limitations discussed: small sample size, non-randomized design, sensor upper-limit constraint, lack of control group |
| Interpretation | 20 | Give a cautious overall interpretation of results considering objectives, limitations, multiplicity of analyses, results from similar studies, and other relevant evidence: **Yes** – Provided a balanced interpretation considering objectives, findings, prior studies, and limitations |
| Generalisability | 21 | Discuss the generalisability (external validity) of the study results: **Yes** - External validity addressed: findings inform real-world ketone supplementation but limited by small sample, young healthy cohort |
| Other information | | |
| Funding | 22 | Give the source of funding and the role of the funders for the present study and, if applicable, for the original study on which the present article is based: **Yes** – Funding and in-kind device support reported (Acknowledgements). |

*Give information separately for exposed and unexposed groups.

**Note:** An Explanation and Elaboration article discusses each checklist item and gives methodological background and published examples of transparent reporting. The STROBE checklist is best used in conjunction with this article (freely available on the Web sites of PLoS Medicine at http://www.plosmedicine.org/, Annals of Internal Medicine at http://www.annals.org/, and Epidemiology at http://www.epidem.com/). Information on the STROBE Initiative is available at http://www.strobe-statement.org.
