# Supplementary material for: Pharmacokinetics and metabolic effects of ketone monoester supplementation: The first simultaneous CKM and CGM study under normal diet and activities
Source: Metabol Open. 2025 Oct 29;28:100411. doi: 10.1016/j.metop.2025.100411 (PMC12617643; doi:10.1016/j.metop.2025.100411)
Supplement: Multimedia component 2 [file mmc2.docx]

**Supplemental Materials**

**Table S1**. Complete list of correlations that had p<.10 from the correlational analysis relating CKM pharmacokinetics features, Oura features, CGM features, DXA and demographic variables, and ASA24 variables.

| **Metric 1** | **Metric 2** | **r** | **n** | **p** |
| --- | --- | --- | --- | --- |
| KME_Tmax | bone_mass | 0.543 | 16 | 0.068 |
| KME_Tmax | bmc | 0.547 | 16 | 0.066 |
| KME_Cmax | KME_onset_time | -0.729 | 16 | 0.007 |
| KME_Cmax | KME_AUC | 0.691 | 16 | 0.013 |
| KME_Cmax | KME_half_life | 0.498 | 16 | 0.099 |
| KME_Cmax | KME_glucose_supression_magnitude | 0.606 | 16 | 0.037 |
| KME_Cmax | weight | -0.555 | 16 | 0.061 |
| KME_Cmax | fat_mass | -0.504 | 16 | 0.095 |
| KME_Cmax | bone_scan_area | -0.536 | 16 | 0.072 |
| KME_onset_time | KME_Cmax | -0.729 | 16 | 0.007 |
| KME_onset_time | KME_glucose_supression_magnitude | -0.731 | 16 | 0.007 |
| KME_onset_time | bone_mass | 0.525 | 16 | 0.08 |
| KME_onset_time | bmc | 0.515 | 16 | 0.087 |
| KME_offset_time | KME_AUC | 0.901 | 16 | <.001 |
| KME_offset_time | KME_half_life | 0.96 | 16 | <.001 |
| KME_offset_time | KME_glucose_supression_time | 0.584 | 16 | 0.046 |
| KME_offset_time | activity_score | -0.624 | 16 | 0.03 |
| KME_offset_time | Average Glucose | -0.561 | 16 | 0.058 |
| KME_offset_time | Glucose Management Indicator (GMI) | -0.577 | 16 | 0.049 |
| KME_offset_time | Target Glucose Range (70-180 mg/dL) | -0.737 | 16 | 0.006 |
| KME_AUC | KME_Cmax | 0.691 | 16 | 0.013 |
| KME_AUC | KME_offset_time | 0.901 | 16 | <.001 |
| KME_AUC | KME_half_life | 0.944 | 16 | <.001 |
| KME_AUC | KME_glucose_supression_magnitude | 0.503 | 16 | 0.096 |
| KME_AUC | KME_glucose_supression_time | 0.518 | 16 | 0.085 |
| KME_AUC | Target Glucose Range (70-180 mg/dL) | -0.714 | 16 | 0.009 |
| KME_half_life | KME_Cmax | 0.498 | 16 | 0.099 |
| KME_half_life | KME_offset_time | 0.96 | 16 | <.001 |
| KME_half_life | KME_AUC | 0.944 | 16 | <.001 |
| KME_half_life | KME_glucose_supression_time | 0.557 | 16 | 0.06 |
| KME_half_life | activity_score | -0.584 | 16 | 0.046 |
| KME_half_life | avg_resting_hr | 0.549 | 16 | 0.064 |
| KME_half_life | Average Glucose | -0.61 | 16 | 0.035 |
| KME_half_life | Glucose Management Indicator (GMI) | -0.622 | 16 | 0.031 |
| KME_half_life | Target Glucose Range (70-180 mg/dL) | -0.761 | 16 | 0.004 |
| KME_glucose_supression_magnitude | KME_Cmax | 0.606 | 16 | 0.037 |
| KME_glucose_supression_magnitude | KME_onset_time | -0.731 | 16 | 0.007 |
| KME_glucose_supression_magnitude | KME_AUC | 0.503 | 16 | 0.096 |
| KME_glucose_supression_magnitude | sleep_score | 0.579 | 16 | 0.049 |
| KME_glucose_supression_magnitude | prot_kcal_ratio | 0.505 | 16 | 0.094 |
| KME_glucose_supression_time | KME_offset_time | 0.584 | 16 | 0.046 |
| KME_glucose_supression_time | KME_AUC | 0.518 | 16 | 0.085 |
| KME_glucose_supression_time | KME_half_life | 0.557 | 16 | 0.06 |
| KME_glucose_supression_time | activity_score | -0.519 | 16 | 0.084 |
| KME_glucose_supression_time | bone_scan_area | 0.514 | 16 | 0.087 |
| KME_glucose_supression_time | Target Glucose Range (70-180 mg/dL) | -0.633 | 16 | 0.027 |
| sleep_duration_hours | sleep_score | 0.841 | 16 | 0.001 |
| sleep_duration_hours | readiness_score | 0.846 | 16 | 0.001 |
| sleep_duration_hours | lean_mass | -0.525 | 16 | 0.08 |
| sleep_duration_hours | tfat_kcal_ratio | -0.551 | 16 | 0.063 |
| sleep_score | KME_glucose_supression_magnitude | 0.579 | 16 | 0.049 |
| sleep_score | sleep_duration_hours | 0.841 | 16 | 0.001 |
| sleep_score | readiness_score | 0.805 | 16 | 0.002 |
| hrv_average | activity_score | -0.583 | 16 | 0.046 |
| hrv_average | fat_mass | -0.537 | 16 | 0.072 |
| hrv_average | tissue_pfat | -0.63 | 16 | 0.028 |
| readiness_score | sleep_duration_hours | 0.846 | 16 | 0.001 |
| readiness_score | sleep_score | 0.805 | 16 | 0.002 |
| readiness_score | lean_mass | -0.571 | 16 | 0.053 |
| readiness_score | tissue_area | -0.517 | 16 | 0.086 |
| readiness_score | bone_scan_area | -0.541 | 16 | 0.069 |
| readiness_score | tfat_kcal_ratio | -0.562 | 16 | 0.057 |
| daily_movement | steps | 0.979 | 16 | <.001 |
| daily_movement | activity_score | 0.879 | 16 | <.001 |
| daily_movement | exercise_frequency | 0.651 | 16 | 0.022 |
| daily_movement | age | 0.9 | 16 | <.001 |
| steps | daily_movement | 0.979 | 16 | <.001 |
| steps | inactive | -0.513 | 16 | 0.088 |
| steps | activity_score | 0.904 | 16 | <.001 |
| steps | exercise_frequency | 0.559 | 16 | 0.059 |
| steps | age | 0.908 | 16 | <.001 |
| inactive | steps | -0.513 | 16 | 0.088 |
| inactive | activity_score | -0.554 | 16 | 0.061 |
| inactive | bone_scan_area | 0.519 | 16 | 0.084 |
| inactive | Average Glucose | -0.514 | 16 | 0.088 |
| inactive | Glucose Management Indicator (GMI) | -0.525 | 16 | 0.08 |
| inactive | Target Glucose Range (70-180 mg/dL) | -0.515 | 16 | 0.087 |
| activity_score | KME_offset_time | -0.624 | 16 | 0.03 |
| activity_score | KME_half_life | -0.584 | 16 | 0.046 |
| activity_score | KME_glucose_supression_time | -0.519 | 16 | 0.084 |
| activity_score | hrv_average | -0.583 | 16 | 0.046 |
| activity_score | daily_movement | 0.879 | 16 | <.001 |
| activity_score | steps | 0.904 | 16 | <.001 |
| activity_score | inactive | -0.554 | 16 | 0.061 |
| activity_score | exercise_frequency | 0.518 | 16 | 0.085 |
| activity_score | age | 0.882 | 16 | <.001 |
| avg_resting_hr | KME_half_life | 0.549 | 16 | 0.064 |
| avg_resting_hr | tissue_pfat | 0.737 | 16 | 0.006 |
| avg_resting_hr | Average Glucose | -0.625 | 16 | 0.03 |
| avg_resting_hr | Glucose Management Indicator (GMI) | -0.603 | 16 | 0.038 |
| avg_resting_hr | Target Glucose Range (70-180 mg/dL) | -0.557 | 16 | 0.06 |
| exercise_frequency | daily_movement | 0.651 | 16 | 0.022 |
| exercise_frequency | steps | 0.559 | 16 | 0.059 |
| exercise_frequency | activity_score | 0.518 | 16 | 0.085 |
| exercise_frequency | tissue_pfat | -0.563 | 16 | 0.057 |
| exercise_frequency | age | 0.63 | 16 | 0.028 |
| exercise_frequency | Average Glucose | 0.556 | 16 | 0.06 |
| exercise_frequency | Glucose Management Indicator (GMI) | 0.628 | 16 | 0.029 |
| exercise_frequency | prot | 0.618 | 16 | 0.032 |
| exercise_frequency | prot_kcal_ratio | 0.511 | 16 | 0.09 |
| weight | KME_Cmax | -0.555 | 16 | 0.061 |
| weight | bone_mass | 0.872 | 16 | <.001 |
| weight | fat_mass | 0.809 | 16 | 0.001 |
| weight | lean_mass | 0.847 | 16 | 0.001 |
| weight | tissue_area | 0.961 | 16 | <.001 |
| weight | bmd | 0.72 | 16 | 0.008 |
| weight | bmc | 0.872 | 16 | <.001 |
| weight | bone_scan_area | 0.773 | 16 | 0.003 |
| height | bone_mass | 0.711 | 16 | 0.009 |
| height | lean_mass | 0.589 | 16 | 0.044 |
| height | tissue_area | 0.647 | 16 | 0.023 |
| height | bmc | 0.698 | 16 | 0.012 |
| height | bone_scan_area | 0.831 | 16 | 0.001 |
| bone_mass | KME_Tmax | 0.543 | 16 | 0.068 |
| bone_mass | KME_onset_time | 0.525 | 16 | 0.08 |
| bone_mass | weight | 0.872 | 16 | <.001 |
| bone_mass | height | 0.711 | 16 | 0.009 |
| bone_mass | lean_mass | 0.958 | 16 | <.001 |
| bone_mass | tissue_area | 0.891 | 16 | <.001 |
| bone_mass | bmd | 0.831 | 16 | 0.001 |
| bone_mass | bmc | 0.999 | 16 | <.001 |
| bone_mass | bone_scan_area | 0.888 | 16 | <.001 |
| bone_mass | kcal | 0.508 | 16 | 0.092 |
| bone_mass | prot | 0.613 | 16 | 0.034 |
| bone_mass | tfat | 0.508 | 16 | 0.092 |
| fat_mass | KME_Cmax | -0.504 | 16 | 0.095 |
| fat_mass | hrv_average | -0.537 | 16 | 0.072 |
| fat_mass | weight | 0.809 | 16 | 0.001 |
| fat_mass | tissue_pfat | 0.797 | 16 | 0.002 |
| fat_mass | tissue_area | 0.728 | 16 | 0.007 |
| fat_mass | Glucose Management Indicator (GMI) | -0.532 | 16 | 0.075 |
| lean_mass | sleep_duration_hours | -0.525 | 16 | 0.08 |
| lean_mass | readiness_score | -0.571 | 16 | 0.053 |
| lean_mass | weight | 0.847 | 16 | 0.001 |
| lean_mass | height | 0.589 | 16 | 0.044 |
| lean_mass | bone_mass | 0.958 | 16 | <.001 |
| lean_mass | tissue_area | 0.859 | 16 | <.001 |
| lean_mass | bmd | 0.815 | 16 | 0.001 |
| lean_mass | bmc | 0.965 | 16 | <.001 |
| lean_mass | bone_scan_area | 0.85 | 16 | <.001 |
| lean_mass | kcal | 0.613 | 16 | 0.034 |
| lean_mass | prot | 0.727 | 16 | 0.007 |
| lean_mass | tfat | 0.607 | 16 | 0.037 |
| tissue_pfat | hrv_average | -0.63 | 16 | 0.028 |
| tissue_pfat | avg_resting_hr | 0.737 | 16 | 0.006 |
| tissue_pfat | exercise_frequency | -0.563 | 16 | 0.057 |
| tissue_pfat | fat_mass | 0.797 | 16 | 0.002 |
| tissue_pfat | Average Glucose | -0.517 | 16 | 0.085 |
| tissue_pfat | Glucose Management Indicator (GMI) | -0.539 | 16 | 0.071 |
| tissue_area | readiness_score | -0.517 | 16 | 0.086 |
| tissue_area | weight | 0.961 | 16 | <.001 |
| tissue_area | height | 0.647 | 16 | 0.023 |
| tissue_area | bone_mass | 0.891 | 16 | <.001 |
| tissue_area | fat_mass | 0.728 | 16 | 0.007 |
| tissue_area | lean_mass | 0.859 | 16 | <.001 |
| tissue_area | bmd | 0.692 | 16 | 0.013 |
| tissue_area | bmc | 0.89 | 16 | <.001 |
| tissue_area | bone_scan_area | 0.824 | 16 | 0.001 |
| tissue_area | tfat | 0.521 | 16 | 0.082 |
| bmd | weight | 0.72 | 16 | 0.008 |
| bmd | bone_mass | 0.831 | 16 | 0.001 |
| bmd | lean_mass | 0.815 | 16 | 0.001 |
| bmd | tissue_area | 0.692 | 16 | 0.013 |
| bmd | bmc | 0.836 | 16 | 0.001 |
| bmd | prot | 0.685 | 16 | 0.014 |
| bmd | prot_kcal_ratio | 0.603 | 16 | 0.038 |
| bmc | KME_Tmax | 0.547 | 16 | 0.066 |
| bmc | KME_onset_time | 0.515 | 16 | 0.087 |
| bmc | weight | 0.872 | 16 | <.001 |
| bmc | height | 0.698 | 16 | 0.012 |
| bmc | bone_mass | 0.999 | 16 | <.001 |
| bmc | lean_mass | 0.965 | 16 | <.001 |
| bmc | tissue_area | 0.89 | 16 | <.001 |
| bmc | bmd | 0.836 | 16 | 0.001 |
| bmc | bone_scan_area | 0.884 | 16 | <.001 |
| bmc | kcal | 0.531 | 16 | 0.076 |
| bmc | prot | 0.632 | 16 | 0.028 |
| bmc | tfat | 0.525 | 16 | 0.079 |
| bone_scan_area | KME_Cmax | -0.536 | 16 | 0.072 |
| bone_scan_area | KME_glucose_supression_time | 0.514 | 16 | 0.087 |
| bone_scan_area | readiness_score | -0.541 | 16 | 0.069 |
| bone_scan_area | inactive | 0.519 | 16 | 0.084 |
| bone_scan_area | weight | 0.773 | 16 | 0.003 |
| bone_scan_area | height | 0.831 | 16 | 0.001 |
| bone_scan_area | bone_mass | 0.888 | 16 | <.001 |
| bone_scan_area | lean_mass | 0.85 | 16 | <.001 |
| bone_scan_area | tissue_area | 0.824 | 16 | 0.001 |
| bone_scan_area | bmc | 0.884 | 16 | <.001 |
| bone_scan_area | tfat | 0.497 | 16 | 0.1 |
| age | daily_movement | 0.9 | 16 | <.001 |
| age | steps | 0.908 | 16 | <.001 |
| age | activity_score | 0.882 | 16 | <.001 |
| age | exercise_frequency | 0.63 | 16 | 0.028 |
| age | Average Glucose | 0.528 | 16 | 0.078 |
| age | Glucose Management Indicator (GMI) | 0.54 | 16 | 0.07 |
| Average Glucose | KME_offset_time | -0.561 | 16 | 0.058 |
| Average Glucose | KME_half_life | -0.61 | 16 | 0.035 |
| Average Glucose | inactive | -0.514 | 16 | 0.088 |
| Average Glucose | avg_resting_hr | -0.625 | 16 | 0.03 |
| Average Glucose | exercise_frequency | 0.556 | 16 | 0.06 |
| Average Glucose | tissue_pfat | -0.517 | 16 | 0.085 |
| Average Glucose | age | 0.528 | 16 | 0.078 |
| Average Glucose | Glucose Management Indicator (GMI) | 0.989 | 16 | <.001 |
| Average Glucose | Target Glucose Range (70-180 mg/dL) | 0.83 | 16 | 0.001 |
| Glucose Management Indicator (GMI) | KME_offset_time | -0.577 | 16 | 0.049 |
| Glucose Management Indicator (GMI) | KME_half_life | -0.622 | 16 | 0.031 |
| Glucose Management Indicator (GMI) | inactive | -0.525 | 16 | 0.08 |
| Glucose Management Indicator (GMI) | avg_resting_hr | -0.603 | 16 | 0.038 |
| Glucose Management Indicator (GMI) | exercise_frequency | 0.628 | 16 | 0.029 |
| Glucose Management Indicator (GMI) | fat_mass | -0.532 | 16 | 0.075 |
| Glucose Management Indicator (GMI) | tissue_pfat | -0.539 | 16 | 0.071 |
| Glucose Management Indicator (GMI) | age | 0.54 | 16 | 0.07 |
| Glucose Management Indicator (GMI) | Average Glucose | 0.989 | 16 | <.001 |
| Glucose Management Indicator (GMI) | Target Glucose Range (70-180 mg/dL) | 0.795 | 16 | 0.002 |
| Glucose Variability | kcal | -0.547 | 16 | 0.065 |
| Glucose Variability | tfat | -0.596 | 16 | 0.041 |
| Target Glucose Range (70-180 mg/dL) | KME_offset_time | -0.737 | 16 | 0.006 |
| Target Glucose Range (70-180 mg/dL) | KME_AUC | -0.714 | 16 | 0.009 |
| Target Glucose Range (70-180 mg/dL) | KME_half_life | -0.761 | 16 | 0.004 |
| Target Glucose Range (70-180 mg/dL) | KME_glucose_supression_time | -0.633 | 16 | 0.027 |
| Target Glucose Range (70-180 mg/dL) | inactive | -0.515 | 16 | 0.087 |
| Target Glucose Range (70-180 mg/dL) | avg_resting_hr | -0.557 | 16 | 0.06 |
| Target Glucose Range (70-180 mg/dL) | Average Glucose | 0.83 | 16 | 0.001 |
| Target Glucose Range (70-180 mg/dL) | Glucose Management Indicator (GMI) | 0.795 | 16 | 0.002 |
| kcal | bone_mass | 0.508 | 16 | 0.092 |
| kcal | lean_mass | 0.613 | 16 | 0.034 |
| kcal | bmc | 0.531 | 16 | 0.076 |
| kcal | Glucose Variability | -0.547 | 16 | 0.065 |
| kcal | prot | 0.863 | 16 | <.001 |
| kcal | tfat | 0.924 | 16 | <.001 |
| kcal | carb | 0.828 | 16 | 0.001 |
| prot | exercise_frequency | 0.618 | 16 | 0.032 |
| prot | bone_mass | 0.613 | 16 | 0.034 |
| prot | lean_mass | 0.727 | 16 | 0.007 |
| prot | bmd | 0.685 | 16 | 0.014 |
| prot | bmc | 0.632 | 16 | 0.028 |
| prot | kcal | 0.863 | 16 | <.001 |
| prot | tfat | 0.795 | 16 | 0.002 |
| prot | carb | 0.615 | 16 | 0.033 |
| tfat | bone_mass | 0.508 | 16 | 0.092 |
| tfat | lean_mass | 0.607 | 16 | 0.037 |
| tfat | tissue_area | 0.521 | 16 | 0.082 |
| tfat | bmc | 0.525 | 16 | 0.079 |
| tfat | bone_scan_area | 0.497 | 16 | 0.1 |
| tfat | Glucose Variability | -0.596 | 16 | 0.041 |
| tfat | kcal | 0.924 | 16 | <.001 |
| tfat | prot | 0.795 | 16 | 0.002 |
| tfat | carb | 0.645 | 16 | 0.023 |
| tfat | tfat_kcal_ratio | 0.654 | 16 | 0.021 |
| carb | kcal | 0.828 | 16 | 0.001 |
| carb | prot | 0.615 | 16 | 0.033 |
| carb | tfat | 0.645 | 16 | 0.023 |
| prot_kcal_ratio | KME_glucose_supression_magnitude | 0.505 | 16 | 0.094 |
| prot_kcal_ratio | exercise_frequency | 0.511 | 16 | 0.09 |
| prot_kcal_ratio | bmd | 0.603 | 16 | 0.038 |
| tfat_kcal_ratio | sleep_duration_hours | -0.551 | 16 | 0.063 |
| tfat_kcal_ratio | readiness_score | -0.562 | 16 | 0.057 |
| tfat_kcal_ratio | tfat | 0.654 | 16 | 0.021 |
| tfat_kcal_ratio | carb_kcal_ratio | -0.591 | 16 | 0.043 |
| carb_kcal_ratio | tfat_kcal_ratio | -0.591 | 16 | 0.043 |

**Figure S1**. All participant’s ISF glucose (blue line) and ketone (black line) levels plotted over 24 hours for each day of the 14-day participation.

**
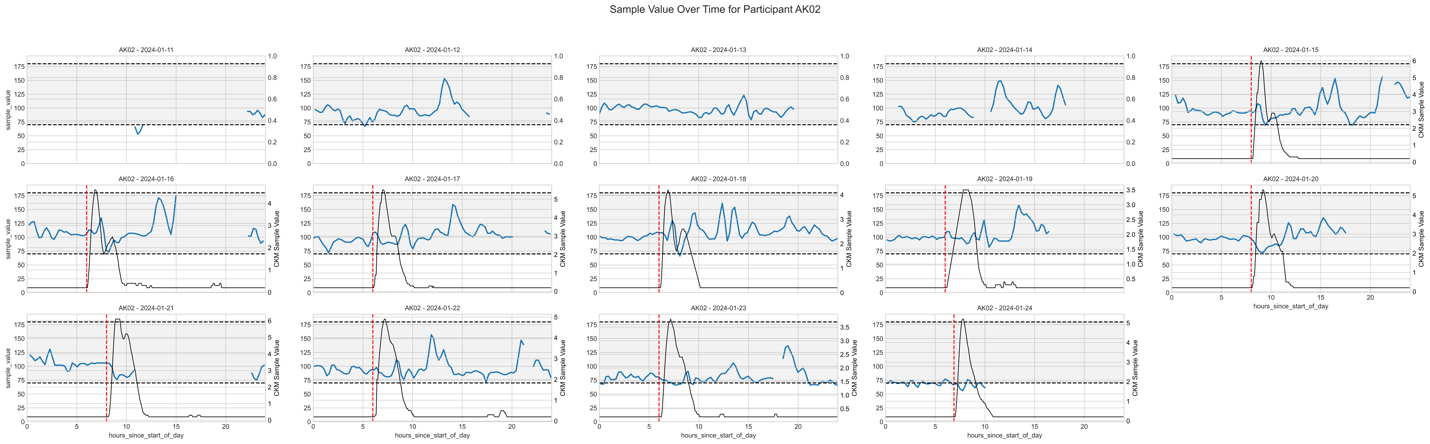

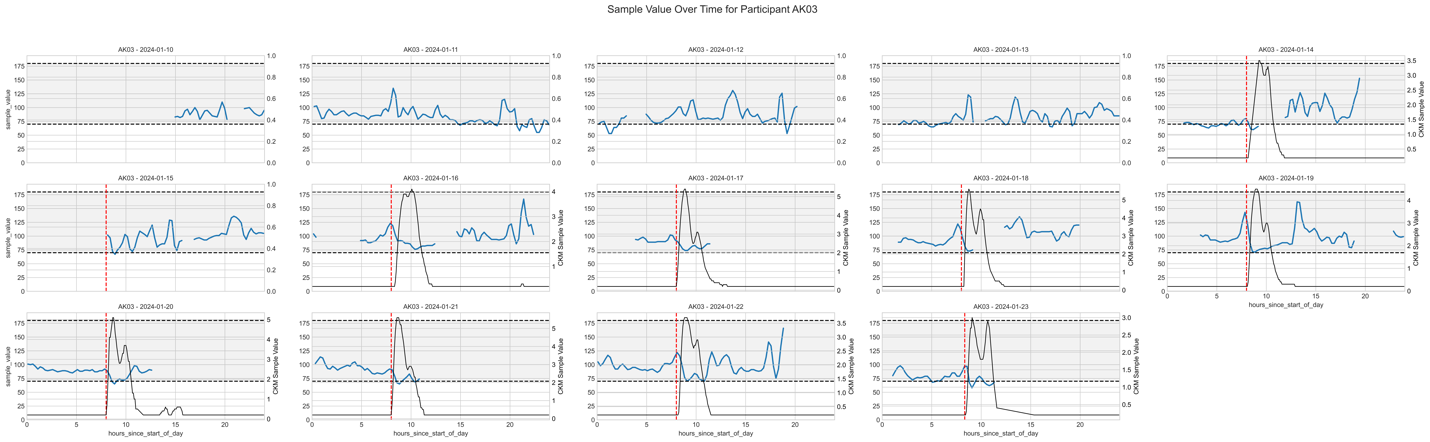

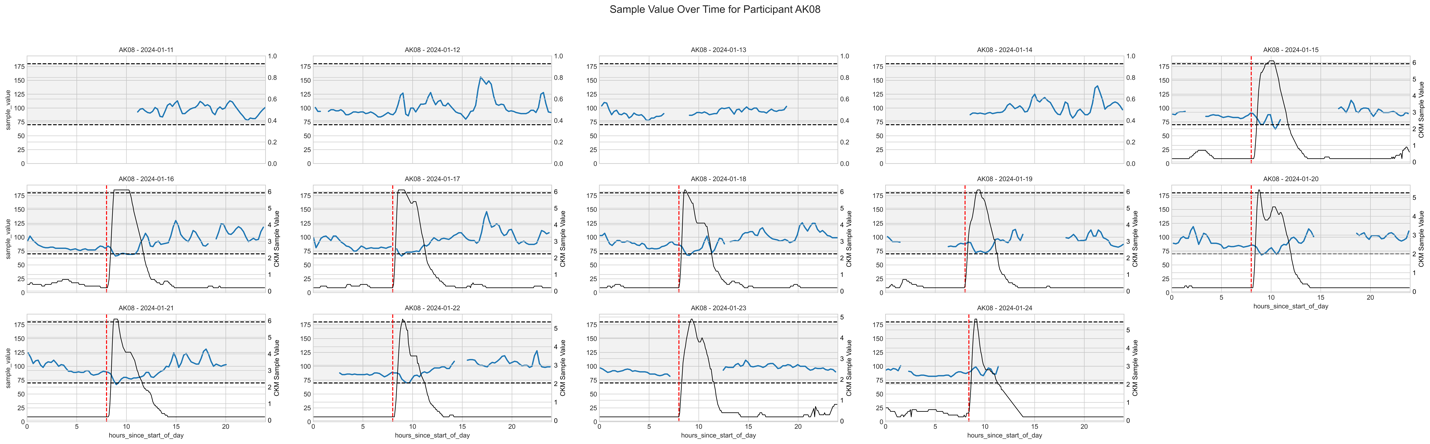

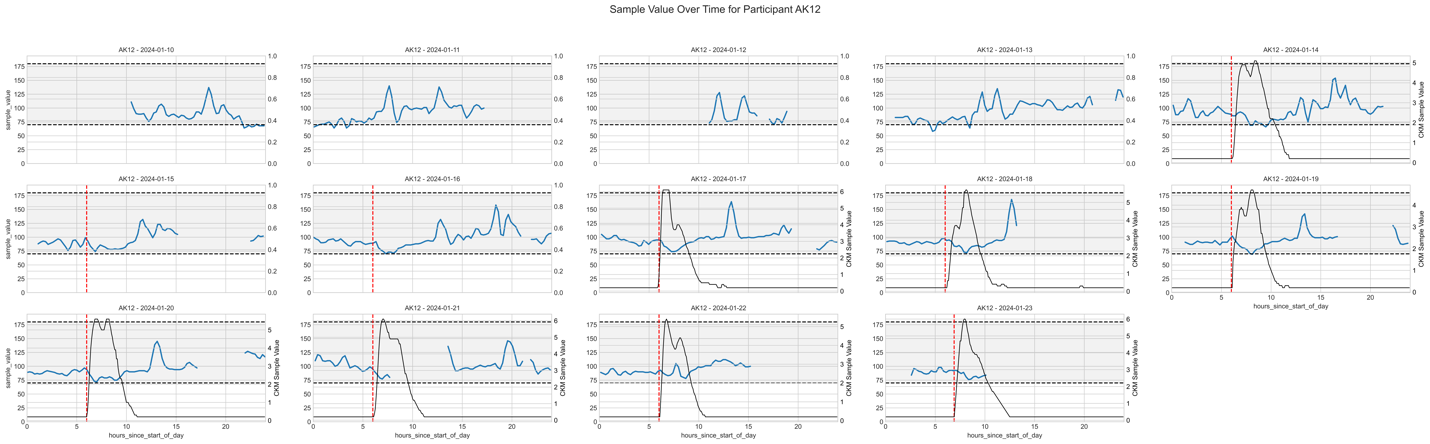

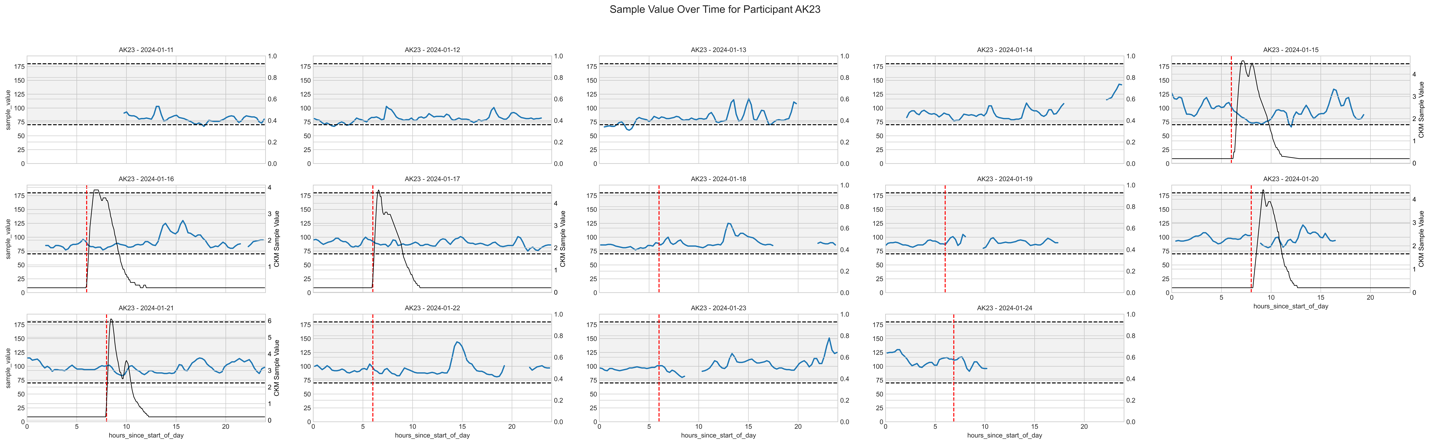

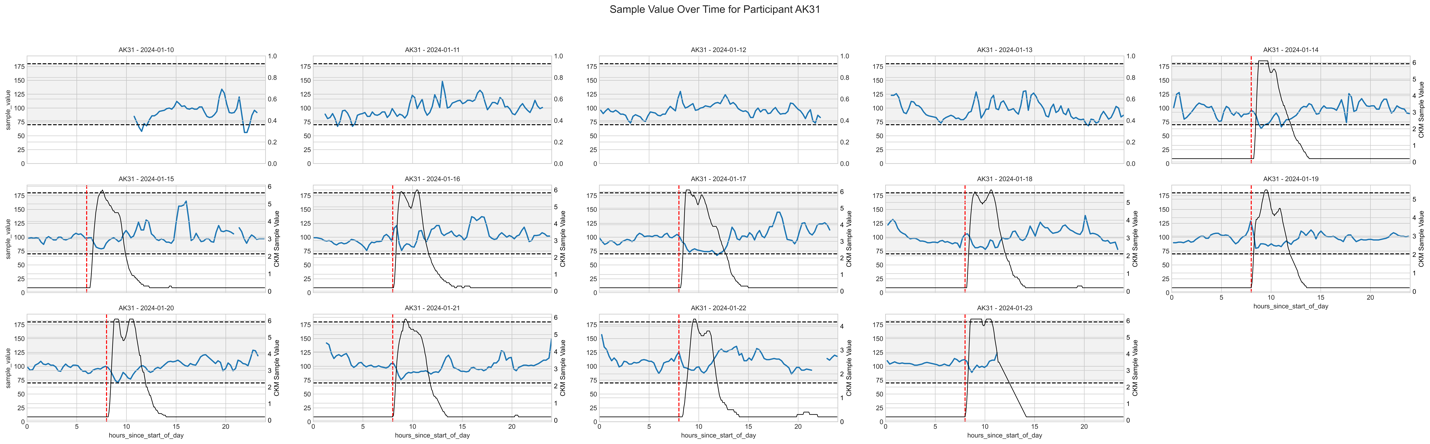

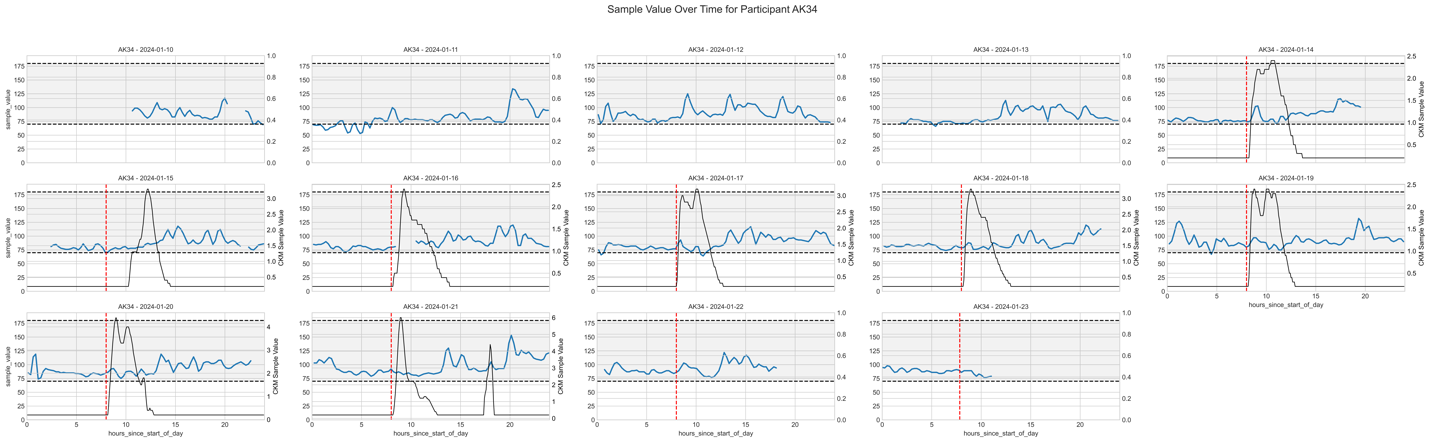

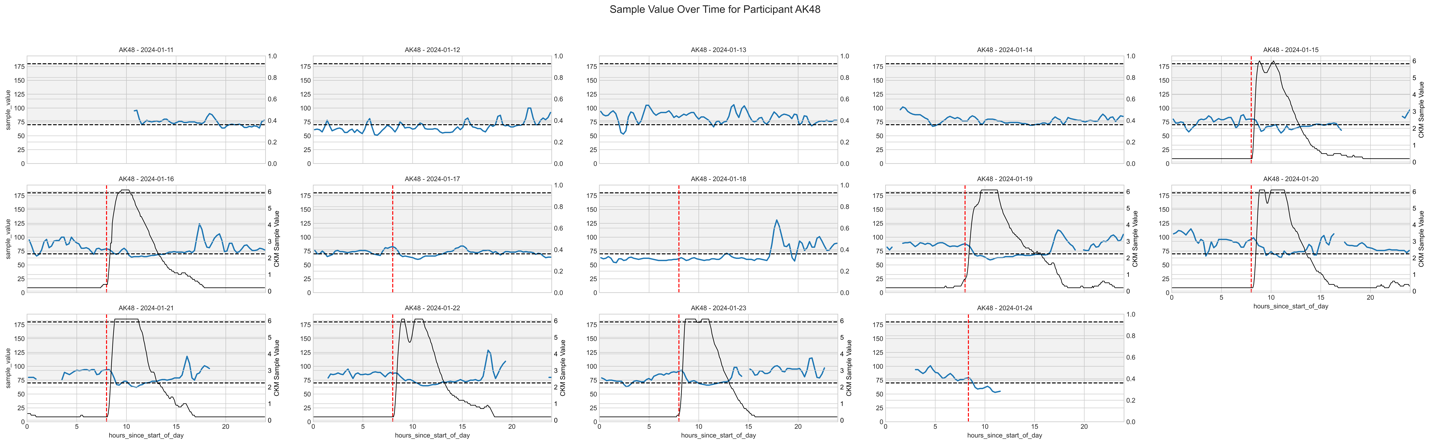

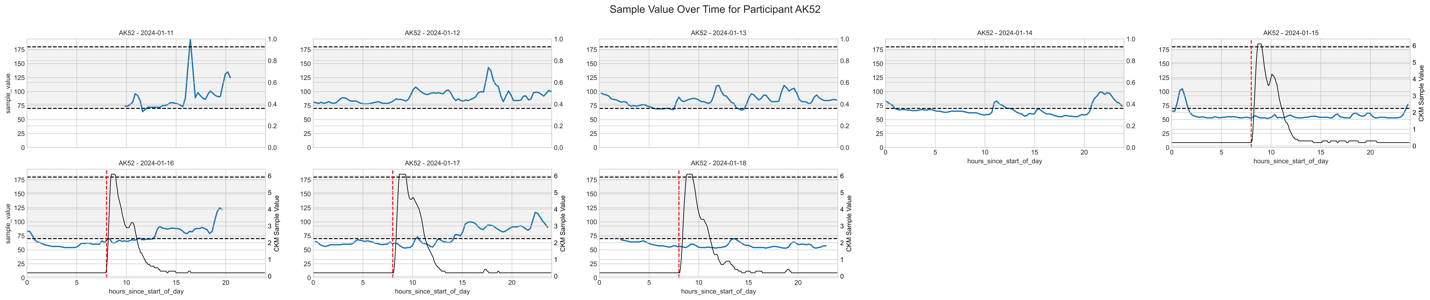

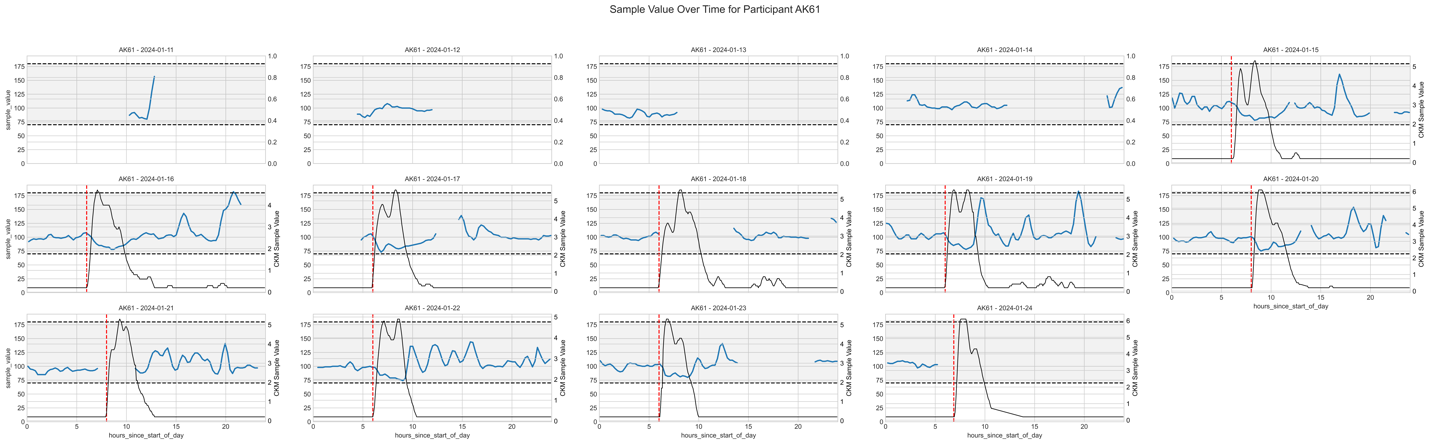

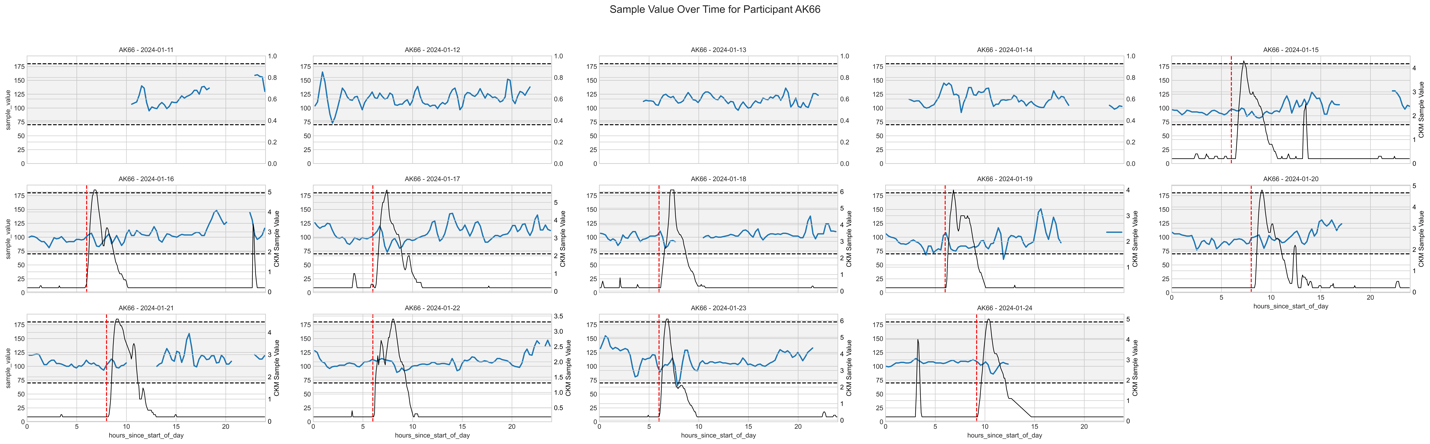

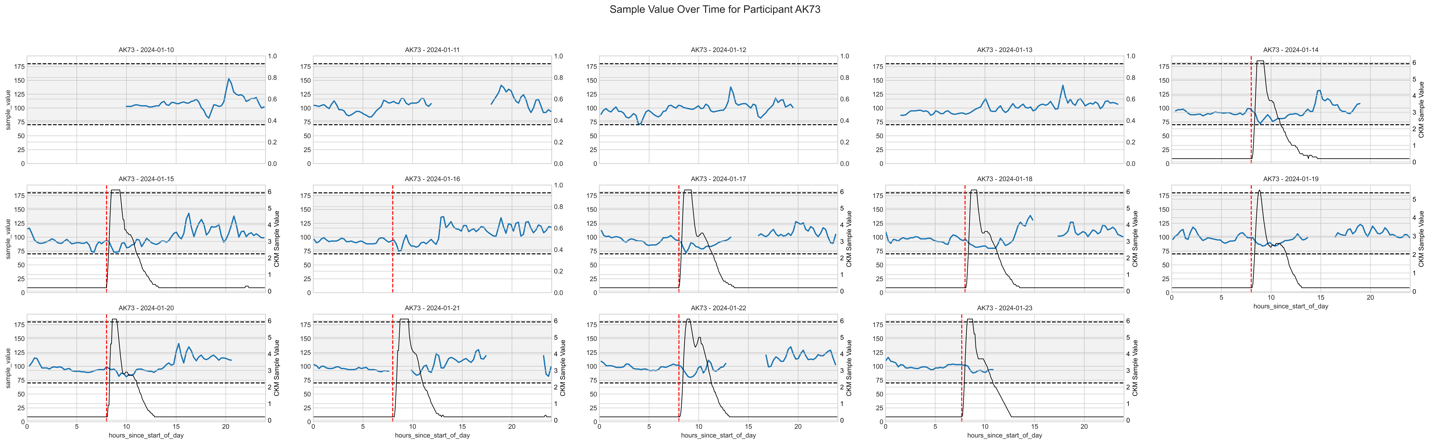

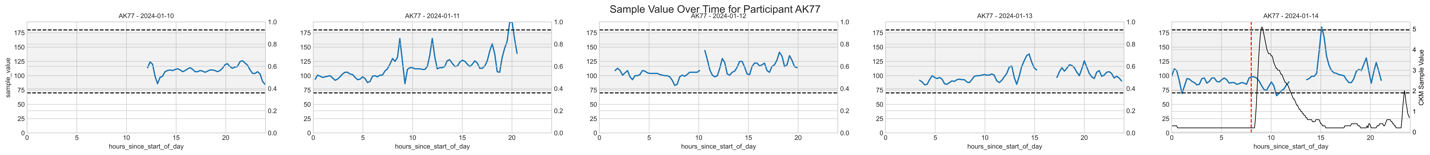

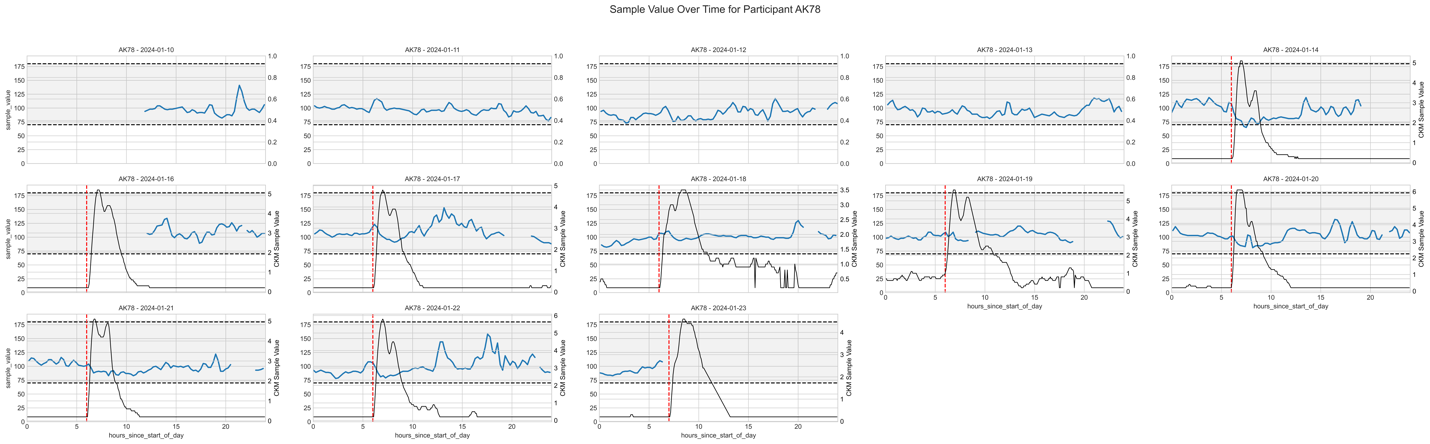

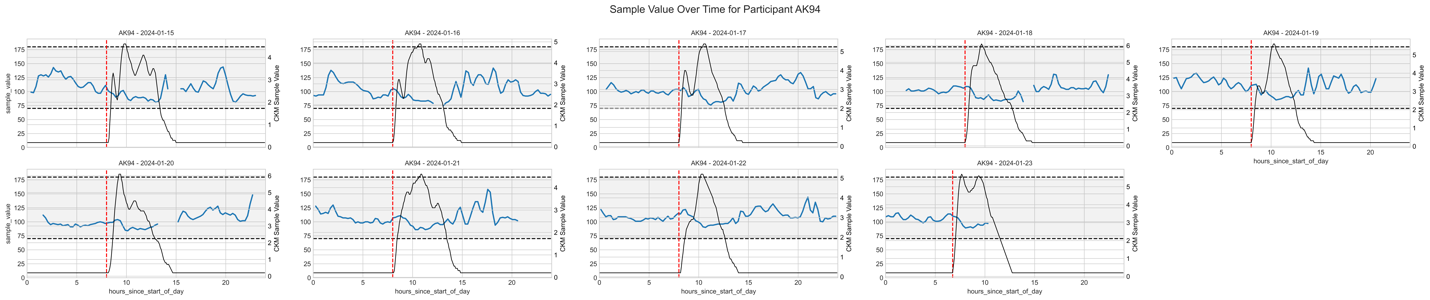

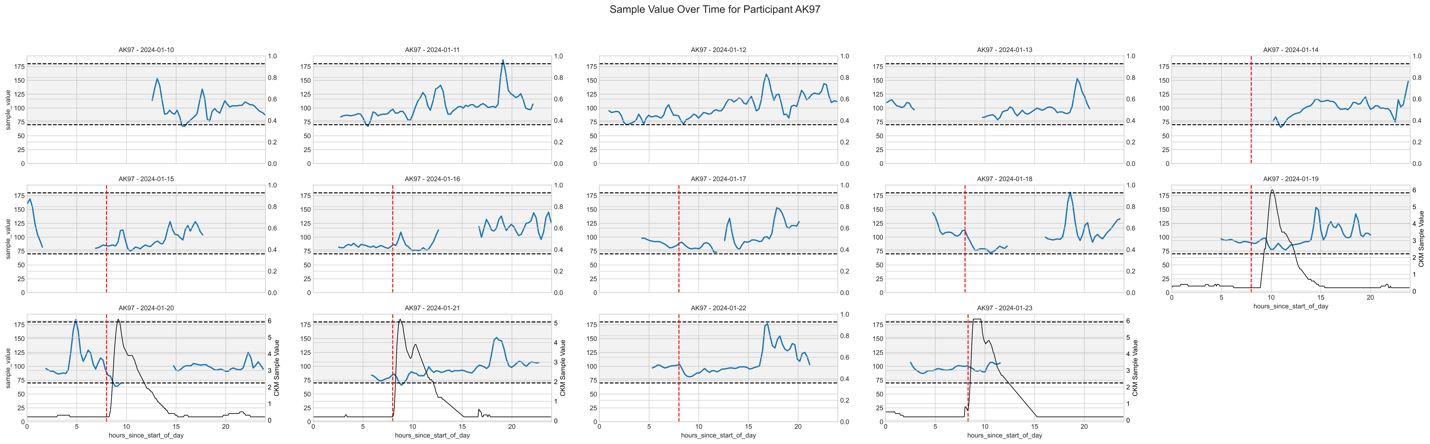
**


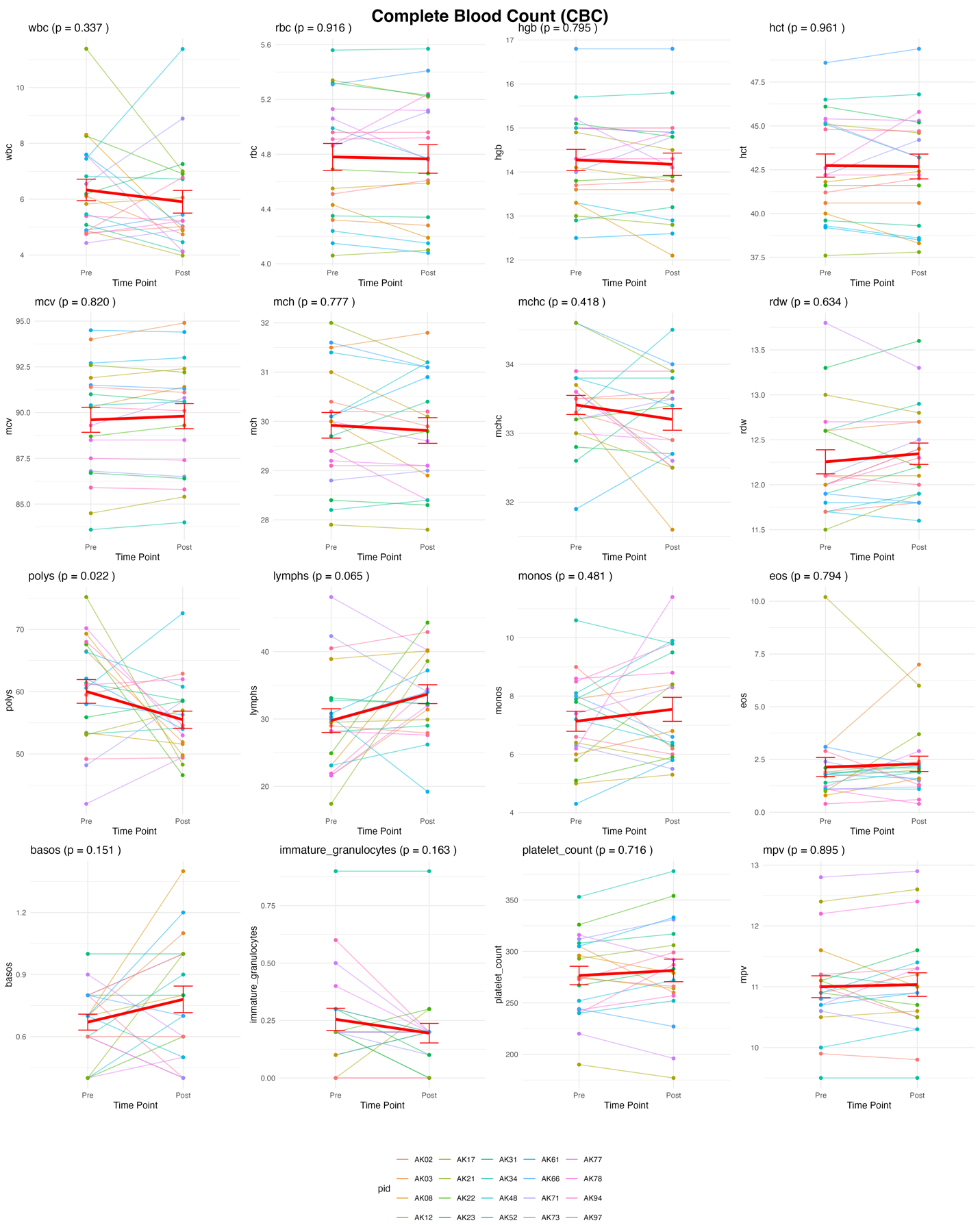


**Figure S2**. Pre-post plots along with the p-values of the paired-sample t-test for all variables from Complete Blood Count (CBC). The red line shows the group mean and other lines show individual participant’s data. The error bars denote the standard error of the mean. wbc: white blood cell count, rbc: red blood cell count, hgb: hemoglobin, hct: hematocrit, mcv: mean


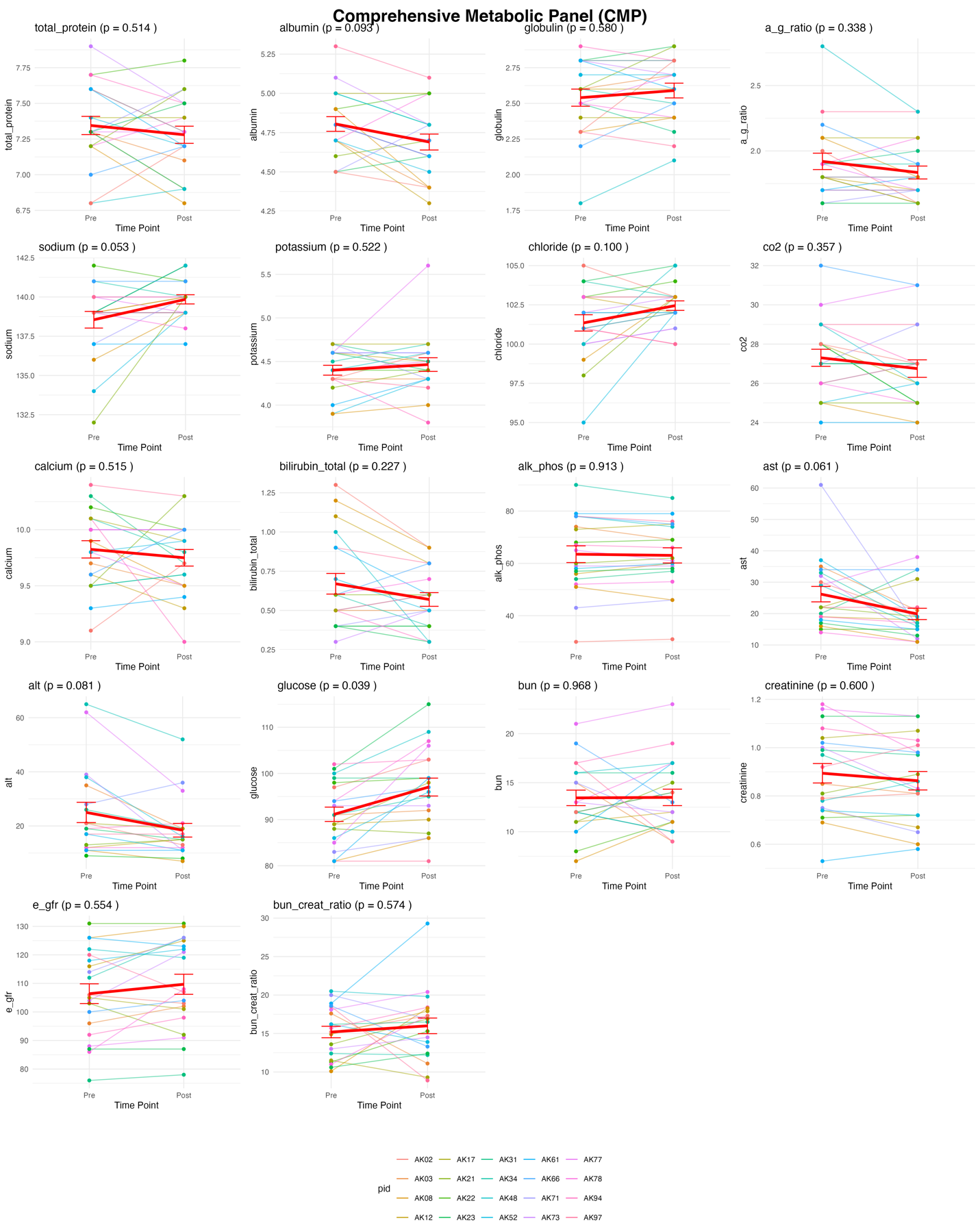


**Figure S3**. Pre-post plots along with the p-values of the paired-sample t-test for all variables from Comprehensive Metabolic Panel (CMP). The red line shows the group mean and other lines show individual participant’s data. The error bars denote the standard error of the mean. total_protein: total protein, albumin: albumin, globulin: globulin, a_g_ratio: albumin to globulin ratio, sodium: sodium, potassium: potassium, chloride: chloride, co2: carbon dioxide, calcium: calcium, bilirubin_total: total bilirubin, alk_phos: alkaline phosphatase, ast: aspartate transaminase, alt: alanine transaminase, glucose: glucose, bun: blood urea nitrogen creatinine: creatinine, e_gfr: estimated glomerular filtration rate, bun_creat_ratio: blood urea nitrogen to creatinine ratio


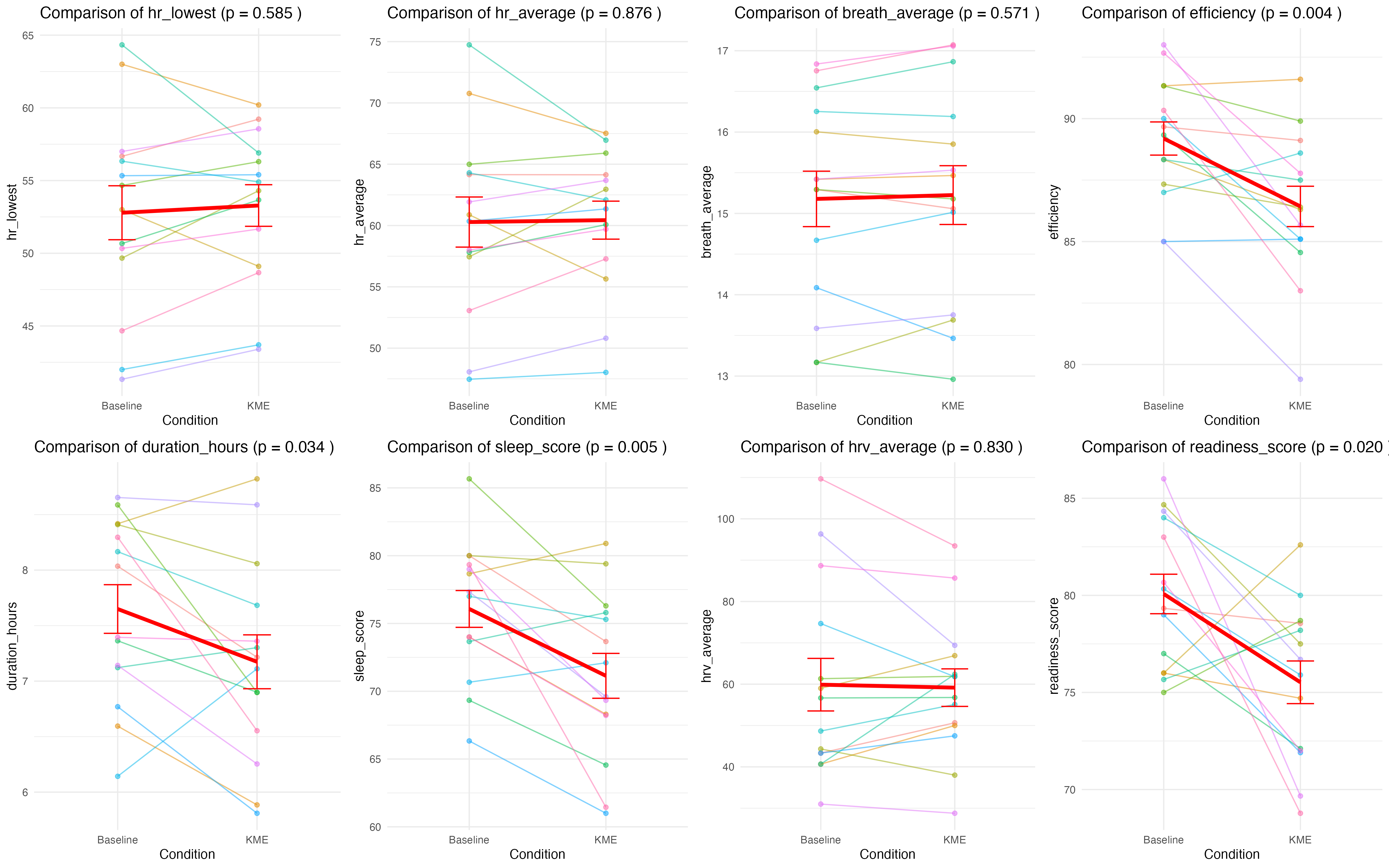


**Figure S4**. Plots comparing Baseline and KME Intervention Phases. The red line shows the group mean and other lines show individual participant’s data. The error bars denote the standard error of the mean. hr_lowest: lowest heart rate during sleep, hr_average: average heart rate during sleep, breath_average: average respiration rate during sleep, efficiency: proportion of time asleep over the time in bed, duration_hours: duration of sleep in hours, sleep_score: Oura’s composite score on sleep, hrv_average: average rmssd during sleep, readiness_score: Oura’s composite score on readiness.
